# Placental Iron Homeostasis: Navigating the Delicate Balance Between Transport and Ferroptosis

**DOI:** 10.3390/ijms27198856

**Published:** 2026-10-04

**Authors:** Dariusz Szukiewicz

**Affiliations:** Department of Biophysics, Physiology & Pathophysiology, Faculty of Health Sciences, Medical University of Warsaw, 02-004 Warsaw, Poland; dariusz.szukiewicz@wum.edu.pl

**Keywords:** iron metabolism, ferroptosis, trophoblast ferroptosis, human placenta, human pregnancy, lipid peroxidation, iron overload, placental dysfunction, Fenton reaction

## Abstract

As an essential micronutrient, iron plays a vital role in oxygen transport, deoxyribonucleic acid synthesis, and energy production and is a redox-active cofactor for many biological processes. However, ferrous iron (Fe^2+^) is toxic to cells because it reacts with hydrogen peroxide in the Fenton reaction to generate the hydroxyl (free) radical, a key player in oxidative stress, leading to lipid peroxidation and iron-dependent cell death (ferroptosis). Therefore, iron homeostasis is precisely regulated. Maintaining appropriate iron metabolism during pregnancy is a major challenge, as it requires adaptation at the level of the utero–placental–fetal unit to the dynamic changes in the demand for iron in the developing fetus during the later weeks of pregnancy. The distinction between physiological (normal) anemia, which results from hemodilution and leads to the depletion of intracellular iron stores (ferritin) in placental cells, and the manifestation of the toxic effects of iron overload and/or oxidative stress is not always clear. Moreover, chronic inflammation during pregnancy associated with increasingly common metabolic disorders such as obesity, diabetes and hypertension may induce ferroptosis. This review presents the current understanding of placental iron homeostasis and its disturbances, focusing on the role of ferroptosis in placental dysfunction. The rationale for potentially effective anti-ferroptotic treatment is also presented.

## 1. Introduction

Iron, abbreviated as Fe (from “ferrum”, the ancient Latin word for the metal), is a crucial micronutrient for almost all living organisms. Despite the recommended dietary allowance (RDA) of iron for humans, which ranges from 8 mg to 18 mg per day and depends on age and biological sex, corresponding to the definition of a trace element (micronutrient), iron plays a disproportionately vital role in the functioning of the body [1,2]. In humans, it supports oxygen metabolism, muscle function, and energy production as a key component of hemoglobin and myoglobin [3,4]. The maintenance of iron homeostasis during pregnancy is particularly important, as confirmed by the recommendations of both the Centers for Disease Control and Prevention and the American College of Obstetricians and Gynecologists, which recommend 27–30 mg of elemental iron as a universal daily oral supplement during pregnancy [5].

Mild iron deficiency anemia, which often accompanies pregnancy, is caused primarily by the body’s drastically increased demand for iron to support the rapidly growing fetus and placenta, along with a 40% to 50% increase in maternal blood volume, which dilutes the existing red blood cell (RBC) count [5,6]. Additionally, it may be caused or exacerbated by nutritional deficits, multiple pregnancies, short intervals between subsequent pregnancies and hyperemesis gravidarum [6,7]. Iron deficiency during pregnancy can lead to severe maternal and fetal complications. Maternal risks include preterm labor, postpartum hemorrhage, cesarean delivery, heart strain and higher rates of postpartum maternal depression [7,8,9]. Fetal and infant risks include low birth weight, neonatal anemia and impaired brain development, particularly altered neurocognitive development with long-term behavioral and mental health challenges [7,8,10,11]. However, maintaining the correct iron balance is critical, as both extremes carry serious risks [12]. Excessive iron or iron overload during pregnancy can be dangerous and is linked to severe complications for both mothers and fetuses, including embryo malformation (embryotoxicity), preeclampsia, gestational diabetes, impaired fetal growth and preterm birth [12,13,14]. The toxic effects of excess iron during pregnancy are directly linked to ferroptosis—an iron-dependent form of regulated cell death driven by overwhelming lipid peroxidation [14,15,16]. Maternal iron overload results in the dysregulation of placental iron transporters, leading to toxic iron accumulation and severe oxidative cellular damage [17,18,19].

Symptoms of disturbances in iron homeostasis during pregnancy can be clearly observed in the placenta because trophoblasts—specifically the multinucleated syncytiotrophoblasts (STB) that line the maternal–fetal interface—are the primary gatekeepers of fetal iron acquisition [20,21]. These cells extract maternal iron, shuttle it via endosomes into fetal circulation, and simultaneously balance placental iron storage and local metabolism [22,23]. Ferroptosis and normal iron homeostasis are maintained in a delicate, dynamic balance [24].

The aim of this narrative review is to present the current understanding of iron metabolism during pregnancy, referring to placental tissue under physiological conditions and considering the involvement of ferroptosis in the pathomechanisms of various pregnancy complications.

## 2. Methodology Used for Literature Search

Through a review of the current literature, a comprehensive analysis of the current state of knowledge on placental iron metabolism in normal and complicated pregnancies was performed, referring to ferroptosis as a consequence of abnormal or dysregulated iron transport leading to cellular iron overload. While trying to maintain maximum criticism and objectivity, a synthesis of relevant research and generalization of the collected data was performed. This approach established a coherent framework for presenting the findings, supporting a qualitative analysis of the topic and facilitating the achievement of the predefined objectives.

Highly cited, peer-reviewed publications in journals from the Master Journal List (MJL) in the fields of anatomy/histology, physiology/pathophysiology, obstetrics, nutrition and dietetics were selected. An important selection criterion for review articles was their topicality (date of publication). The electronic databases PubMed, Scopus, Web of Science (WoS) and Google Scholar were reviewed. Priority was given to studies providing observational or experimental evidence. The core of the literature search consisted of publications within the time frame of 2015–2026, and in the case of review articles with the same topic defined by keywords, publications from journals positioned in Q1 were preferred. During manuscript preparation, the citation list was then supplemented with key milestone articles that captured the phenomena and the biologically active compounds’ properties, cited regardless of publication date.

The general scope of the literature included:

The papers on the general principles of iron metabolism in the body, including iron metabolism during pregnancy, identified using the following leading keywords/phrases: “iron metabolism”, “iron absorption”, “cellular iron transport”, “cellular iron storage”, “systemic iron homeostasis”, “cellular iron regulation”, “intestinal iron uptake”, “erythropoiesis”, “iron recycling (macrophage iron recycling)”;

Sources exploring the relationship between intracellular iron storage disorders and hemochromatosis, hemosiderosis in the context of ferroptosis were chosen from research combining theoretical models with experimental findings. The relevant leading keywords/phrases included “ferroptosis”, “iron overload syndromes”, “labile iron pool”, “Fenton reaction”, “lipid peroxidation”, “iron storage disorders”, “pathomechanisms of hemochromatosis”;

Research data on the role of the placenta in iron metabolism in normal and complicated pregnancy, obtained using the following leading keywords/phrases: “placental iron homeostasis”, “placental iron trafficking”, “trophoblast ferroptosis”, “placental ferroptosis”, “ferroptosis and placental dysfunction”;

The papers providing rationale for recommended remedies and potential therapeutic options to prevent placental ferroptosis-related complications, identified using the following leading keywords/phrases: “ferroptosis inhibitors”, “anti-ferroptotic agents”, “metainflammation and placental ferroptosis”, “iron supplementation during pregnancy and placental ferroptosis”, “SLC7A11-GSH-GPX4 axis”.

Ultimately, this narrative review included 269 publications, including 109 original research papers and 160 review articles.

## 3. General Principles of Iron Metabolism in the Body

### 3.1. Iron Content and Distribution

The average iron content in the adult human body is approximately 42 to 55 milligrams per kilogram of tissue [25,26]. Assuming that for the general adult population in Western countries, the average measured weight is approximately 172 lb (78 kg) for women and 199 lb (90 kg) for men, with an average body mass index (BMI) of 26.6, this translates to a total body iron content of 3.3 to 5.0 g for women and men, respectively [27]. Sex-related differences in iron content stem from the lower erythrocyte mass and iron stores (myoglobin and ferritin) in women owing to their lower muscle mass and the effects of estrogen (see the next section: Estrogen–iron axis) [28,29].

The total iron content of the body is distributed across three main pools: functional, storage and transport [30,31,32].

The functional pool comprises approximately 65% to 75% of the total iron—which is found in the hemoglobin of red blood cells (RBCs) and erythroid precursors as well as in the myoglobin of muscle cells—and is primarily involved in oxygen transport (hemoglobin) and the formation of intracellular oxygen reservoirs (myoglobin) [33]. The remaining trace amounts of iron within the functional pool, sometimes referred to as the enzymatic protein pool, are integrated into the cytochromes and iron-sulfur clusters necessary for cellular respiration and adenosine 5′-triphosphate (ATP) generation at the mitochondrial level [34,35]. Iron is also found in catalases—i.e., antioxidative enzymes that convert hydrogen peroxide into water and oxygen to protect cells from oxidative damage—which are composed of four subunits containing heme (iron-porphyrin) as a prosthetic group [36,37].

The storage pool, also known as the reserve pool, comprises 20% to 30% of the total iron in the body, approximately 0.8 g of which is stored in liver parenchymal cells, primarily in the form of ferritin [38]. This water-soluble, spherical protein safely stores excess iron in its core to prevent cellular toxicity [39]. In states of significant hepatocyte iron overload—where the amount of excess iron exceeds the cells’ capacity to safely store the trace element in a soluble form—it is also stored as hemosiderin, an insoluble iron-storage complex derived from degraded ferritin within lysosomes [40,41]. In addition to hepatocytes, reticuloendothelial macrophages in the spleen, liver, and bone marrow—which are involved in iron transport—serve as iron reserves [42,43].

The transport (transit) pool of iron consists primarily of the glycoprotein transferrin, which binds to circulating iron in the bloodstream [44]. By binding virtually all plasma iron produced by the liver, transferrin protects cells from the high toxicity of free iron and allows for its safe transport from intestinal cells and macrophages to primarily bone marrow cells for erythropoiesis [45,46]. The iron transport system operates via a highly dynamic, recycling-based process, and despite the small amount of the transferrin pool, which comprises approximately 3 mg to 4 mg of iron in circulation, the daily iron turnover reaches 20 mg to 25 mg [47].

In addition, a small fraction of transported iron—typically constituting less than 5% of total cellular iron—serves as a transient, intracellular reservoir of loosely bound, redox-active iron [48]. Because ferrous iron reacts with hydrogen peroxide, which serves as a catalyst to generate highly reactive cell-damaging hydroxyl radicals via the Fenton reaction, its levels are strictly regulated by intracellular proteins [49]. Dysregulation of this pool is closely linked to severe conditions, such as oxidative stress, ferroptosis (see Section 5.3. Iron overload and placental ferroptosis) and cancer [50].

The iron content and distribution in the human body are presented in Table 1.

### 3.2. Intestinal Iron Absorption

Strict control of daily intestinal iron absorption by systemic hormones and local cellular mechanisms is particularly important because the human body does not have an active, regulated mechanism to excrete excess iron [52].

**The iron regulatory protein/iron responsive element (IRP/IRE) system** acts as the primary cellular and basal regulator of intestinal iron absorption by determining how much iron is imported, stored, or exported by absorptive cells in the duodenum [53,54]. This regulation occurs through post-transcriptional management of translation and stability of key iron transporter and storage mRNAs in duodenal enterocytes [55,56]. IRP1 and IRP2 are cytosolic RNA-binding proteins that sense intracellular iron levels and bind to IREs [57]. IREs, in turn, are specific hairpin structures found in the untranslated regions (UTRs) of target mRNAs [58,59]. When IRPs bind to an IRE in the 5′-UTR of an mRNA (such as ferritin or ferroportin), they block translation, reducing the production of these proteins [60]. Conversely, when IRPs bind to an IRE in the 3′-UTR of an mRNA (such as DMT1 or transferrin receptor 1), they protect the transcript from degradation, increasing protein expression by mRNA stabilization [61].

**Hepcidin**, a hormone produced in the liver, serves as the main systemic regulator; by binding to the iron exporter ferroportin [also known as solute carrier family 40 member 1 (SLC40A1)] on the basolateral membrane of duodenal enterocytes, it triggers its degradation, thereby inhibiting the transfer of iron into the bloodstream [62]. Hepcidin production increases owing to elevated systemic iron levels and inflammation, actively limiting iron absorption to prevent toxicity [62,63]. Conversely, during iron deficiency or increased erythropoiesis, hepcidin production decreases, allowing ferroportin to remain stable and export more iron into circulation [51].

Mucosal iron sensing involves the expression of the transcription factor **hypoxia-inducible factor 2-alpha (HIF-2α)** in enterocytes, which responds directly to intracellular iron and oxygen levels [64]. In low-oxygen or systemic iron-deficient states, intestinal HIF-2 α is stabilized and binds directly to HREs on genes for key iron transporters at the apical membrane, including divalent metal-ion transporter 1 (DMT1) and the enzyme duodenal cytochrome b (DcytB) [64,65,66]. The reduction and transport of dietary Fe^3+^ into enterocytes are accelerated. Additionally, HIF-2α directly increases the transcription of the ferroportin (FPN/SLC40A1) gene, which leads to an adaptive increase in ferroportin protein expression and enhanced iron export (efflux) from duodenal enterocytes into the bloodstream [67]. In an indirect mechanism, via the renal-hepatic axis, HIF-2α leads to a reduction in the production of hepcidin [64]. This is because the main function of HIF-2α is to strongly activate erythropoietin (EPO) production in the kidneys and liver. Increased EPO concentrations stimulate erythropoiesis in the bone marrow, leading to the secretion of erythroferrone (ERFE) by erythroblasts. ERFE then reaches the liver and inhibits the bone morphogenic protein/small mother against decapentaplegic (BMP/SMAD) pathway, lowering hepcidin levels to free iron stores for hemoglobin production [68,69,70]. Low hepcidin levels allow upregulated ferroportin to remain open and active for a prolonged duration.

HIF-2α is also involved in another mammalian iron-sensing axis via the IRP and IRE machinery [71]. This is due to the presence of a stem-loop structure hidden within the HIF2α mRNA within its 5′ untranslated region (UTR), corresponding to the IRE [72,73,74]. This suggests the existence of a further iron- and oxygen-dependent mechanism for translational regulation of HIF-2α.

Non-heme iron (Fe^3+^) absorption is strongly influenced by dietary factors and is usually low, ranging from 2 to 20%, while the food components have a minor effect on heme iron (Fe^2+^) from meat, the bioavailability of which is significantly higher and amounts to 15 to 35% [75,76]. The mechanism of intestinal absorption of heme iron differs from that of non-heme iron primarily in that heme is taken up as an intact metalloporphyrin complex, whereas non-heme iron requires chemical reduction [77]. Heme iron therefore uses a separate, specialized pathway to enter the intestinal enterocyte intact. This apical uptake is mediated by heme carrier protein 1 (HCP1) and potentially heme responsive gene-1 (HRG1) or via receptor-mediated endocytosis [76,78,79]. Inside the cell, under the influence of heme oxygenase-1 (HO-1), enzymatic cleavage of the porphyrin ring of the heme molecule occurs [80]. Fe^2+^ is liberated, which joins the common pool with iron from the non-heme pathway, and bilirubin, which can be removed from the cell by the efflux proteins feline leukemia virus subgroup C cellular receptor (FLVCR) and ATP-binding cassette subfamily G member 2 (ABCG2, also known as breast cancer resistance protein (BCRP)) [78,81].

**Heavy chain ferritin (H-ferritin)** plays a critical role in preventing iron overload and limiting iron basolateral efflux into the bloodstream [82]. H-ferritin possesses ferroxidase activity, rapidly converting toxic ferrous iron (Fe^2+^) into safe, stored ferric iron (Fe^3+^) within the enterocytes. Therefore, intestinal H-ferritin acts as a local brake by trapping absorbed dietary iron inside the mucosal cells. Since hepcidin-mediated regulation alone is not sufficient to limit iron absorption, H-ferritin participates in a key mechanism of fine-tuning, in parallel with hepcidin-related regulation [82,83].

### 3.3. Iron Transport and Utilization

Divalent iron (ferrous ion, Fe^2+^), which is transported out of enterocytes into the bloodstream by ferroportin, is immediately oxidized in the plasma by a ferroxidase enzyme (such as hephaestin or ceruloplasmin) into the trivalent form (ferric ion, Fe^3+^), which can bind to transferrin [24]. Each transferrin molecule can safely carry up to two iron atoms, and iron-loaded transferrin (holo-transferrin; holo-Tf) delivers iron to all cells possessing transferrin receptors (primarily TfR1) on their surface. The entire holo-Tf/TfR1 complex is internalized via endocytosis [84]. Intracellular iron is released owing to the high acidity of the endosome interior, causing the iron to dissociate from transferrin; it is then transported into the cell’s cytoplasm to be used or stored in ferritin. Transferrin carries iron mainly to the bone marrow, where it is utilized to produce mature RBCs (hemoglobin) [85]. The remainder of the iron is delivered to tissues with high metabolic needs, such as the liver and spleen, for storage and systemic distribution. Iron-free transferrin (apo-transferrin; apo-Tf) remains bound to TfR1 and is recycled back to the cell surface, where it is subsequently released into the bloodstream [44].

Cellular iron homeostasis is regulated by posttranscriptional feedback mechanisms, which control the expression of proteins involved in iron uptake, release and storage mainly through two cytoplasmic proteins with mRNA-binding properties, iron regulatory proteins 1 and 2 (IRP1 and IRP2) [86]. By binding to specific noncoding sequences, known as iron-responsive elements (IREs), within the untranslated regions (UTRs) of mRNA transcripts, IRP1 (90 kDa) and IRP2 (105 kDa) form a system that regulates ferritin translation and thus iron storage. For example, when intracellular iron levels are low, IRPs bind to IREs within the 5′ UTR of ferritin mRNA, resulting in reduced translation rates [87]. Additionally, through mRNA stabilization, IRP/IRE interactions increase TfR1 expression and iron uptake via TfR1-mediated endocytosis of iron-loaded transferrin [88]. PCBP1 (poly(rC)-binding protein 1) is a vital cytosolic iron chaperone that safely binds and delivers iron to key intracellular targets such as ferritin, iron-dependent enzymes, iron-sulfur cluster assembly complexes and ferroportin. It prevents cellular toxicity by controlling iron reactivity [65].

### 3.4. Storage and Its Disorders

Ferritin is a 24-subunit protein composed of two types of chains: heavy (H) and light (L). As a storage protein, ferritin safely encapsulates up to 4500 iron atoms, which remain readily available when erythropoiesis increases in the body [89]. Hemosiderin, which forms stable protein–iron complexes, serves as a long-term iron reservoir, particularly when iron levels become significantly (and dangerously) elevated [89]. The physiological profiles of iron storage and distribution exhibit sex-related differences. Adult males typically store approximately 1000 mg of iron in intracellular reserves, whereas menstruating females often have storages closer to 500 mg because of higher iron turnover [90,91].

Disorders associated with iron overload are known as hemochromatosis and hemosiderosis [92,93]. Hemochromatosis involves excessive systemic iron absorption and deposition within the parenchymal cells of organs such as the liver, heart, and pancreas, leading to organ damage; this can be caused by genetic factors (usually a mutation of the homeostatic iron regulator (HFE) gene) or occur as a secondary reaction because of frequent blood transfusions or severe chronic liver disease [94]. In hemochromatosis, iron is deposited in amounts exceeding the cell’s normal ferritin storage capacity, resulting in a substantial pool of labile (free) intracellular iron, leading to toxic effects such as ferroptosis [95,96].

The primary causes of hemosiderosis, i.e., a focal or systemic accumulation of hemosiderin, include localized bleeding, chronic hemolysis and repeated blood transfusions [97]. Iron deposits are located primarily within tissue-resident macrophages of the mononuclear phagocyte system (MPS) [98]. Unlike hemochromatosis, hemosiderosis typically does not cause structural or functional damage to affected organs because the excess iron is safely bound and sequestered inside hemosiderin; thus, it cannot easily participate in the Fenton reaction to generate reactive oxygen species (ROS). As a result, the molecular triggers for ferroptosis are largely avoided [89].

### 3.5. Recycling and Excretion

Over 90% of the body’s iron stores undergo recycling, and the small amount of iron lost daily—on the order of 1–2 mg—is replenished through intestinal absorption during the digestion of nutrients containing bioavailable iron [4]. The primary source of iron recovery is the degradation of the hemoglobin heme group, which occurs as a result of the destruction of aging erythrocytes by specialized reticuloendothelial macrophages in the spleen and liver in a process termed erythrophagocytosis. The iron released from the macrophage phagolysosome is transported into the cytosol via natural resistance-associated macrophage protein 1 (NRAMP1), a divalent metal transporter and paralog of DMT1 [99]. Afterward, the liberated iron can be stored in ferritin or released back into systemic circulation by ferroportin-mediated transport across the plasma membrane, and after binding with transferrin in plasma, it reaches the bone marrow to synthesize new RBCs [100]. Heme is transported from the phagolysosome to the cytoplasm by heme responsive gene 1 (HRG1), an essential transmembrane protein for macrophage iron homeostasis. HRG1 is strongly expressed in macrophages of the reticuloendothelial system and specifically localizes to the phagolysosomal membranes during erythrophagocytosis [101]. Heme in the cytoplasm can be degraded by inducible form of heme oxygenase 1 (HO-1), a catabolic enzyme whose action creates the rate-limiting step in the oxidative breakdown of heme into three primary products: biliverdin, carbon monoxide (CO), and free ferrous iron (Fe^2+^) [102,103,104,105].

A key role in iron metabolism is played by liver sinusoidal endothelial cells (LSECs), highly specialized cells lining the tiny blood vessels, which act as the body’s main iron sensors by producing bone morphogenetic protein 6 (BMP6), the signaling protein regulating systemic iron balance [106]. LSECs detect changes in blood iron levels, including non-transferrin-bound iron and hemoglobin [106,107]. LSECs are characterized by the presence of open pores called fenestrae without diaphragms, the lack of a structured basement membrane, and an exceptionally high capacity for endocytosis [108,109]. They have the highest permeability of any mammalian endothelial cell [108,110]. When the level of iron in the blood increases, LSECs release BMP6 into the narrow gap in their environment, the space of Disse (also called the perisinusoidal space), which transmits a signal to hepatocytes in a paracrine manner [111]. Binding of BMP6 to hepatocyte receptors triggers the production of hepcidin to block ferroportin. Recent research shows LSECs also directly clear hemoglobin from the blood (via a process called macropinocytosis) to recycle iron safely and prevent toxicity during hemolysis [106,107].

The system tightly governing iron absorption, utilization, storage, recycling, and excretion is semiclosed in nature, as the only routes of iron excretion are passive loss via menstrual blood in premenopausal women and the shedding of gastrointestinal epithelial cells and skin cells [52].

**Figure 1:**
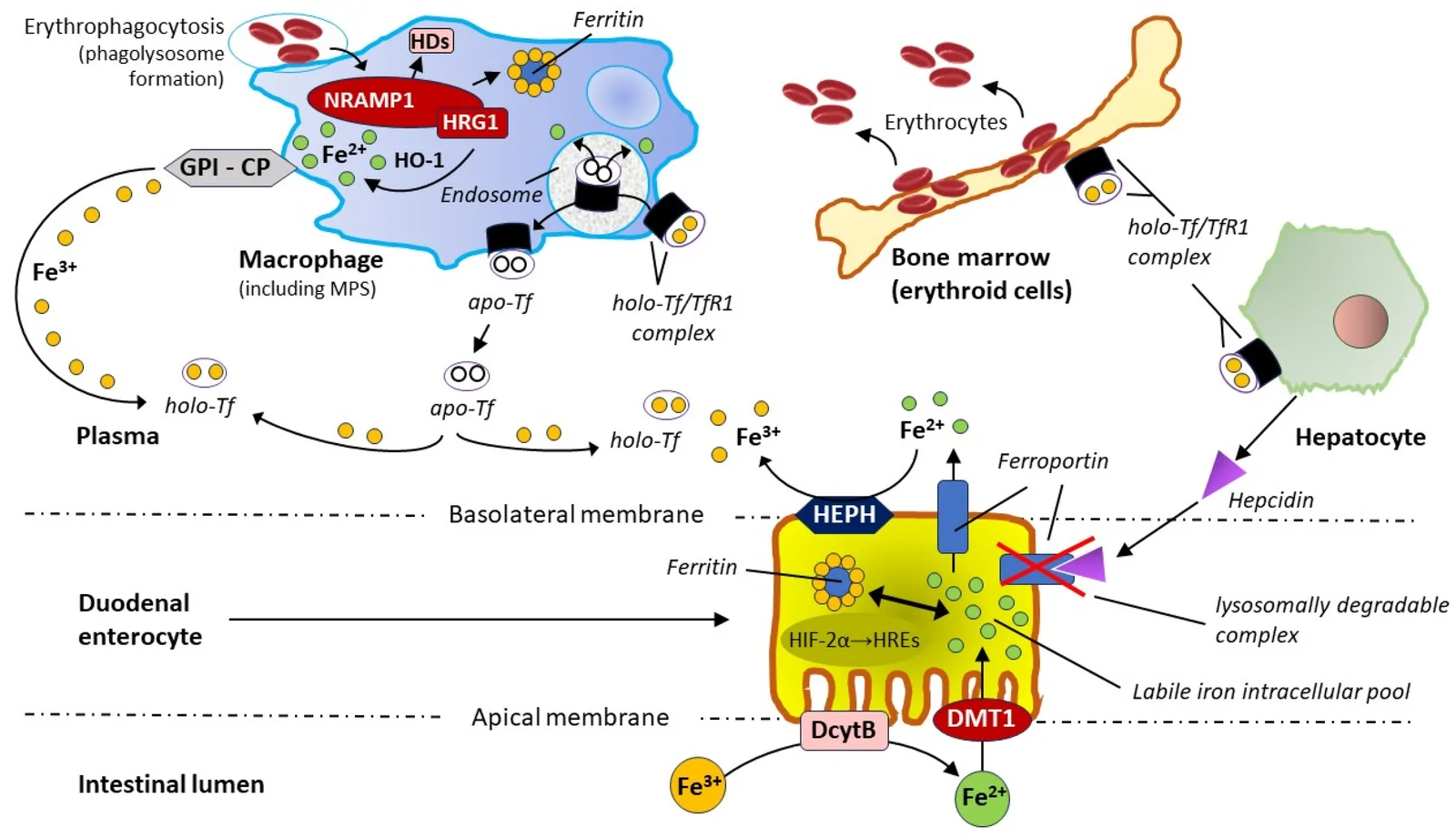
Key components of iron metabolism and homeostasis in the human body [4,62,64,86,91,101,103]. The level of intestinal iron absorption is the main factor ensuring its homeostasis. Low-oxygen or systemic iron-deficiency states increase the expression and distribution of hypoxia-inducible factor 2-alpha (HIF-2α) in enterocytes, which binds to hypoxia-response elements (HREs) on the genes encoding major iron transporters at the apical membrane, such as DMT1 and the enzyme duodenal cytochrome b (DcytB). Iron as a divalent ion enters the cytoplasm, where it forms a labile pool or—in trivalent form—is stored as ferritin. Ferroportin enables the transport of iron through the basolateral membrane into the blood, where the divalent form is oxidized by ferroxidases [hephaestin (HEPH) or ceruloplasmin (CP)] to the trivalent form, which binds to the transport protein transferrin. CP capable of physically interacting with ferroportin occurs attached to the outer surface of the macrophage membrane via a glycosylphosphatidylinositol (GPI) anchor, which is abbreviated as GPI-CP. Holo-transferrin (holo-Tf), whose molecule transfers up to two iron atoms, reaches all cells expressing surface transferrin receptors, mainly the type 1 receptor (TfR1). A holo-Tf/TfR1 complex is formed and is fully endocytosed. Following intracellular iron release, iron-free transferrin or apo-transferrin (apo-Tf) remains bound to TfR1 and is recycled back to the cell surface with subsequent release into the bloodstream. Transferrin transports iron primarily to erythrocyte lineage cells in the bone marrow for hemoglobin production and delivers the remaining iron to the liver and macrophages of the mononuclear phagocyte system (MPS) in the spleen for storage and systemic distribution. Aging RBCs are removed from the blood primarily by macrophages via erythrophagocytosis. The phagolysosomes of macrophages contain natural resistance-associated macrophage protein 1 (NRAMP1), a protein essential for efficiently extracting and recycling iron from the hemoglobin inside the engulfed RBCs back into the body’s circulation or ensuring storage in the form of ferritin or—especially in the case of risk of toxic iron overload—hemosiderin deposits (HDs). Heme responsive gene 1 (HRG1) is essential for heme transport from the phagolysosome of macrophages to the cytoplasm, where it is catabolized by inducible form of heme oxygenase 1 (HO-1); the resulting divalent iron is mainly exported by ferroportin or stored in ferritin as Fe^3+^. Hepatocytes produce hepcidin, a hormone that controls the access of iron to the bloodstream. By binding to ferroportin on the surface of enterocytes, macrophages and hepatocytes (for clarity, only enterocytes are shown in the figure), hepcidin causes lysosomal degradation of the formed complex, which completely blocks the release of iron into the blood.

The main factors contributing to iron metabolism and ensuring iron homeostasis are summarized in Figure 1.

### 3.6. Estrogen—Iron Axis

The regulatory mechanism based on the physiological feedback loop that allows estrogen to influence iron metabolism is known as the estrogen–iron axis [112]. Clinical and experimental evidence suggests that periods with increased estrogen levels (e.g., the ovulatory phase of the menstrual cycle, pregnancy) are characterized by more efficient dietary iron absorption from the gut and maximum systemic iron bioavailability [113,114]. The estrogen–iron axis has been shown to act through both direct (through estrogen response elements (EREs)) and indirect (e.g., through G-protein-coupled estrogen receptor 1 (GPR30) and bone morphogenetic protein 6 (BMP6) pathways) mechanisms in duodenal enterocytes, macrophages and hepatocytes [115,116]. Estrogen suppresses hepcidin and limits ferroportin mRNA transcription in estrogen receptor-positive (ER^+^) cells. In this way, E_2_ prevents ferroportin degradation, and because this effect dominates the reduction in ferroportin mRNA transcription, intracellular iron (ferritin) stored in duodenal enterocytes, macrophages and hepatocytes is redistributed into the plasma [115]. This increase in systemic iron availability to meet hematologic (erythropoiesis) and nonhematologic demands is particularly important during pregnancy and is essential for uterine growth and placental and fetal development [21,23,117]. However, the direct involvement of pregnancy-related hormones, including those in the estrogen–iron axis, in the mechanism of hepcidin suppression during pregnancy remains uncertain and requires further investigation [21,114].

The effects of the estrogen–iron axis can be reversed in states characterized by low estrogen levels (e.g., during menopause and in certain hormonal disorders associated with amenorrhea), where iron absorption is restricted, accompanied by an increased risk of localized iron deposition or deficiency [118,119]. Thus, while the activity of the estrogen–iron axis helps compensate for the monthly blood loss that occurs in premenopausal women, in postmenopausal women using hormone replacement therapy, it may increase susceptibility to iron overload if not monitored [120,121].

## 4. Iron Metabolism and Pregnancy

Adaptive Changes in Iron Metabolism During Pregnancy.

Of the approximately 1000 mg of additional iron required by the mother during pregnancy, ~300 mg is transferred to the fetus and placenta, ~500 mg is involved in the increase in the number of maternal red blood cell mass, and ~200 mg is lost through normal bodily excretion (e.g., exfoliation of dead skin, blood loss and turnover of the intestinal epithelium) [21]. The temporary cessation of menstrual bleeding during pregnancy allows only approximately 200–300 mg of iron to be preserved [122]. Avoiding a massive imbalance between iron supply and demand during pregnancy requires many adaptive changes, with hepcidin inhibition playing a key role. The strong inhibition of hepcidin synthesis often results in undetectable levels in the third trimester of pregnancy [114]. Hepcidin suppression occurs through the maximization of dietary iron absorption, which is particularly pronounced from week 24 of gestation until term, and the increased release of the intracellularly stored iron into the bloodstream [123]. The active transport of nonheme iron across the apical membrane of duodenal enterocytes is increased because of increased local expression of DMT1; moreover, the lack of hepcidin prevents the degradation of ferroportin at the level of the basolateral membrane, which increases the export of ferrous iron from the inside of cells such as enterocytes, macrophages and hepatocytes [124,125].

The mechanisms of pregnancy-related hepcidin suppression are not fully understood, and recent studies have not confirmed the previously suggested direct effect of elevated estradiol and prolactin levels during pregnancy [21,114]. Currently, when the causes of the reduced hepcidin concentration are analyzed, three key factors are considered:

The natural decrease in blood iron levels and the depletion of iron reserves in hepatocytes in a pregnant woman’s body—resulting from the uptake of significant amounts of iron by the placenta and the growing fetus—directly inhibit hepcidin synthesis in the liver [21,126].

The increased iron demand driven by intensified erythropoiesis—associated with the significant increase in circulating blood volume during pregnancy—acts as a bone marrow-derived signal that suppresses hepcidin; however, this excludes the erythroid-derived hepcidin-suppressing factor erythroferrone (ERFE) [127,128].

An increase in the serum concentration of the soluble form of hemojuvelin (sHJV) during gestation—which acts as a decoy receptor within the bone morphogenetic protein (BMP)/small mother against decapentaplegic (SMAD) family–hepcidin axis—inhibits BMP signaling and reduces hepcidin expression [129,130,131].

Rising estrogen levels during pregnancy stimulate the liver to increase the synthesis of transferrin, which increases total iron-binding capacity (TIBC) in the blood [132]. This adaptive change serves as a compensatory mechanism to meet the increased iron needs of both the mother and the developing fetus. While the average serum transferrin concentration is in the range of 200–400 mg/dL (2.5–5.0 μmol/L) in nonpregnant women, the concentration of this transport protein gradually increases and reaches values in the range of 288–530 mg/dL (3.5–6.5 μmol/L) during the third trimester [133,134,135]. However, despite the increase in the total amount of iron in the body, transferrin saturation (iron saturation) tends to decrease slightly during pregnancy because of the disproportionate increase in blood volume (hemodilution) [21,136,137]. This may be misinterpreted as iron deficiency.

Dynamic increases in estrogen levels during pregnancy transcriptionally upregulate the expression of lactoferrin through specific EREs located within the lactoferrin gene promoter. Lactoferrin is a specialized multifunctional protein belonging to the transferrin family that binds iron [138]. The increase in plasma lactoferrin levels during pregnancy is accompanied by its presence in breast milk, with particularly high concentrations found in colostrum [138,139]. The core functions of lactoferrin include immune support through antimicrobial, antiviral, and anti-inflammatory activities; iron regulation to ensure its safe absorption in the body; antioxidant action; and the modulation of coagulation and hemostasis during pregnancy [138,139]. Lactoferrin, which is upregulated by estrogen, is responsible for physiological pregnancy-associated hypercoagulability by interacting with and potentiating factor XIa (FXIa), an active blood plasma enzyme that facilitates the production of blood clots [140]. While this physiological shift promotes hemostasis to mitigate peripartum hemorrhage risk, it also exacerbates maternal hypercoagulability, thereby increasing the incidence of thromboembolic morbidity [140].

## 5. Role of the Placenta in Iron Metabolism

In the hemochorial placenta found in humans and rodents, maternal blood is in direct contact with fetal chorionic villi and trophoblast cells, with no intervening maternal tissue layers [141]. Iron is therefore transferred from maternal circulation via the blood flowing through placental STBs. Iron transport across the placenta is unidirectional and requires cellular energy because it occurs against a concentration gradient [22]. Because iron builds up in the fetus at higher levels than it does in the mother, receptors and channels handle iron intake and release within placental circulation in a way that prioritizes the fetus, which means that iron is acquired by the fetus even when iron is deficient in maternal circulation [22,142]. However, several human and animal studies challenge the classic dogma of exclusive fetal control over the iron transfer process, as compromised fetal iron stores have been demonstrated in cases of severe maternal iron deficiency [8,143]. Thus, maternal and local signals may also be considered in iron regulation.

### 5.1. Normal Placental Iron Transport

Iron transport at the placental level occurs in several precise stages, and its intensity increases as pregnancy progresses [144]. The rate of this increase in iron transport cannot be explained by a simple increase in the number of transferrin receptors (TfRs) due to an increase in their surface area caused by placental growth [144,145]. The most important components (stages and mechanisms) of this process are as follows:

Iron uptake by the placenta occurs through the binding of transferrin to appropriate receptors on the surfaces of trophoblasts [20];

The intracellular release of iron occurs through its detachment from transferrin and, via PCBP1, storage as ferritin and/or subsequent release as a transport ligand/ion for ferroportin [146];

Iron trafficking occurs across STBs and through the fetal endothelium into the fetal blood [127];

At the peak of iron transport, which occurs in the third trimester of pregnancy, the fetus accumulates the greatest reserves of this trace element, which is stored mainly in the liver and used during the first months of life after birth [147].

The villi (and the syncytiotrophoblast microvilli covering them) in the human placenta are directly immersed in the maternal blood located in the intervillous spaces (placental sinuses) [148]. Iron transported in the placenta must travel at the level of the fully developed villi (approximately 18–20 weeks of pregnancy), including two plasma membranes of STBs (the microvillous plasma membrane contacting the maternal blood and the basal membrane facing the stromal core of the villi) [149]. Afterward, iron moves in the connective tissue within the space between the STBs and the fetal vessels, called the villous core stroma (or extraembryonic mesoderm), which contains mesenchymal cells, fibroblasts, and Hofbauer cells (specialized fetal macrophages), toward the fetal capillary endothelium, i.e., the inner lining of the fetal blood vessels, which consists of polarized cells with a luminal surface facing the bloodstream and a basal (basolateral) surface facing the underlying basement membrane and stroma [150,151]. Iron transport across fetal capillary endothelial cells (ECs) relies on regulated transcellular pathways involving specific uptake and efflux mechanisms rather than passive paracellular leakage [20,152].

Several transporters expressed in the abovementioned plasma membranes participate in the transfer of iron across the placenta to the fetal blood, and the intercellular spaces of the stroma of the villi also participate in the regulation of maternal–fetal signals, forming a system of fluid-filled channels containing extracellular vesicles (stromal macrovesicles), among other components [145,153].

The main mechanism of iron transport between maternal and fetal blood involves diferric transferrin [Tf-Fe(III)2], which forms a complex on the apical side of STBs after binding to TfR1 [22]. The expression of both holo-Tf and TfR1 on the apical surface of placental microvilli increases as pregnancy advances and fetal iron demand increases [154]. The key role of transferrin in placental iron transport has been confirmed by studies on hypotransferrinemic mice, whose offspring showed an extremely severe form of iron deficiency anemia (IDA) and survived only up to 2 weeks without transferrin or serum administration [155]. The holo-Tf/TfR1 complex is endocytosed in clathrin-coated vesicles, and the intracellular mechanism for holo-Tf-bound iron processing after endocytosis is similar to that of enterocytes, macrophages and hepatocytes [156]. Because TIBC is higher in maternal blood than in umbilical cord blood, iron transport due to placental transferrin transcytosis may occur only in trace amounts [157]. The decrease in the pH inside the endosome from 7.4 to less than 5.5 results in the release of ferric iron from Tf, which is subsequently reduced to ferrous iron by the ferric reductases of six-transmembrane epithelial antigen of prostate (STEAP) family members, specifically STEAP3 and STEAP4 [21,158]. Both DMT1 and ZRT/IRT-like protein 14 (ZIP14) enable the release of Fe^2+^ from the endosome into the cytoplasm, where it can be stored by ferritin, incorporated into heme and heme-derivative proteins within the placenta, or released into fetal capillaries via ferroportin [159,160]. Similar to hephaestin in the gut, a multicopper ferroxidase, zyklopen, is expressed in the cellular membrane and oxidizes ferrous iron to ferric iron after it is transferred through ferroportin [161,162]. In this way, the exported, less toxic ferric iron can safely bind to fetal transferrin in the placental stroma and passively diffuse (along with free Fe^3+^ ions) in the extracellular fluid over a short distance [20,146]. The loose connective tissue of the placental villous stroma acts as a simple aqueous space, known as the wet stromal matrix, which fills the narrow physical gap between the trophoblast basement membrane and the outer wall of the fetal capillary [153,163]. In addition to the basolateral syncytiotrophoblast membrane, ferroportin is expressed in the core villous stroma cells to transfer iron into the fetal capillary endothelium [163].

The least understood stage of placental iron transfer is the mechanism of movement of this element within the villous stroma toward the capillary endothelium and the subsequent transendothelial transport to fetal blood [20,145]. A “classic mechanism” is assumed in which the diferric fetal Tf complex approaches the fetal capillary endothelium and binds to TfR1 located on the abluminal (outer/stromal-facing) side of the capillary ECs. The holo-Tf/TfR1 complex is subsequently internalized into ECs via receptor-mediated endocytosis. Owing to the acidity of the endosome interior, the ferric ion detaches from Tf and is converted to ferrous iron mainly by STEAP4; then, iron may be stored in the form of ferritin or exported to fetal blood [164]. How iron subsequently traverses the microvascular ECs of the fetal villous capillaries to reach the circulation remains unclear, and this polarized endothelial expression and luminal vs. abluminal distribution must be further investigated [21]. Typically, this polarized placement allows controlled iron transendothelial efflux from the blood into local tissue or the brain interstitium, and ferroportin, the only known cellular iron exporter in vertebrates, is expressed on the abluminal (basolateral) surface of ECs [17,165].

Notably, there is no clear evidence that the dominant, transferrin-related placental transfer of iron to the fetus is the only way for fetal tissues to acquire this element. In mouse studies, disruption of TfR1 alleles significantly reduced, but did not prevent, the generation of substantial numbers of RBCs in embryos, suggesting the existence of an alternative iron uptake mechanism during early embryogenesis [123,145]. Other transferrin-independent iron transport mechanisms may involve primarily heme transporters that are highly expressed in the placenta, including the transmembrane receptor low-density lipoprotein (LDL) receptor-related protein 1 (LRP1) [166], the feline leukemia virus subgroup C receptor 1 (FLVCR1) [167,168], and the proton-coupled folate transporter (PCFT), also known as heme carrier protein 1 (HCP1) [169]. Additionally, iron transport independent of transferrin may occur with the participation of DMT1 and heme oxygenase (HO)-1 and HO-2 [170,171,172]. In pathological conditions associated with elevated transferrin iron saturation, placental transport of non-transferrin-bound iron (NTBI) may occur, which increases the risk of toxic effects if ferrous iron (Fe^2+^) triggers the Fenton reaction [173]. Additionally, zinc transporters belonging to the solute carrier family 39A (known as the ZIP or ZRT- and IRT-like protein family), such as ZIP 8 (SLC39A8) and ZIP 14 (SLC39A14), can transport both non-transferrin-bound and transferrin-bound iron in the placenta [174]. Further studies are needed to determine the role of non-transferrin-mediated placental iron transfer in the physiological and pathophysiological mechanisms of pregnancy at different stages [18,175].

**Figure 2:**
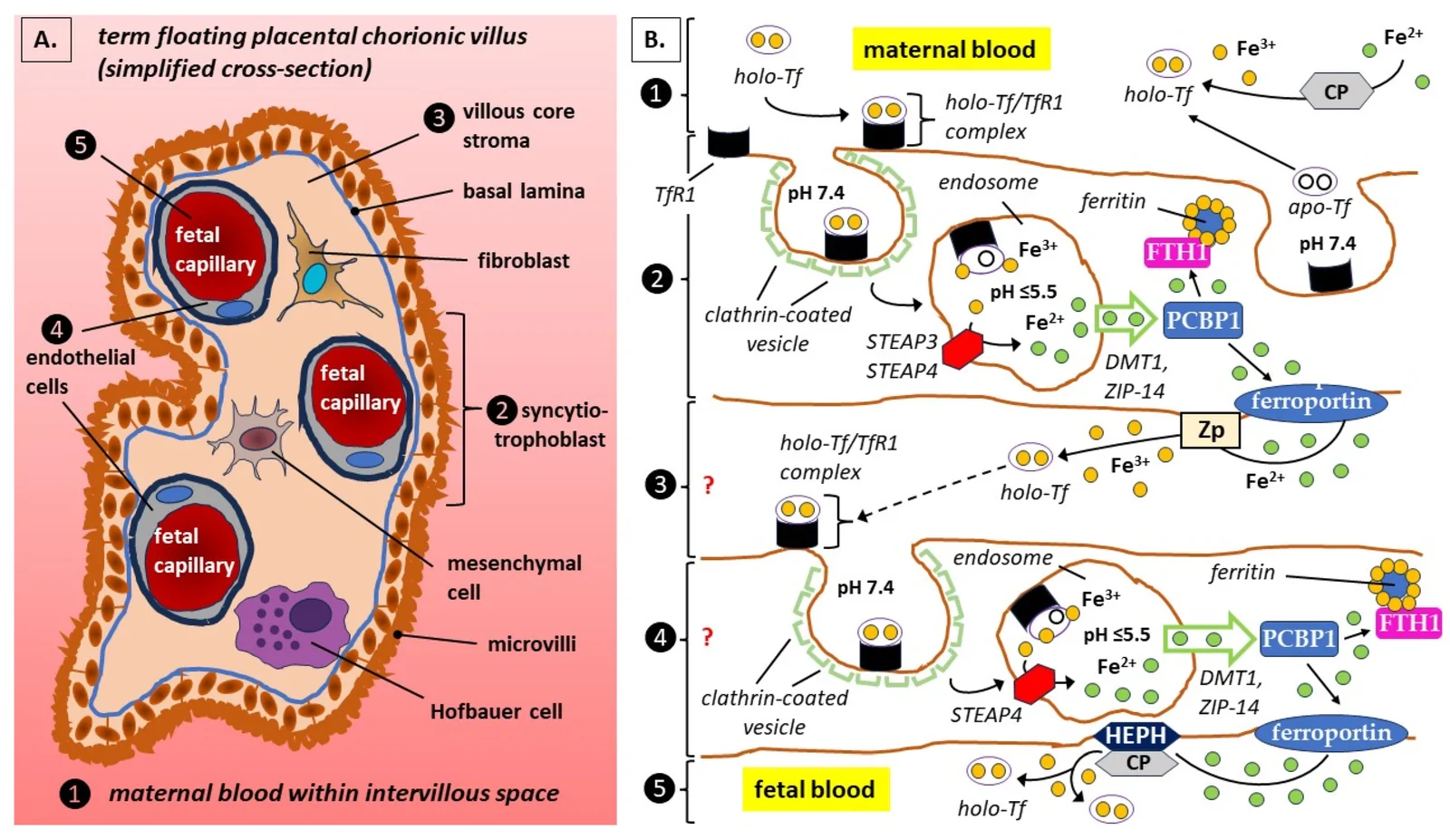
Main mechanism of iron uptake and transport in the human placenta [20,21,22,23,52,145,176]. (**A**) Key structural elements of the placental barrier. (**B**) Five stages (❶ to ❺) of iron trafficking across the placental barrier from maternal blood within intervillous space into the interior of the fetal capillaries. The red question marks accompanying stages 3 and 4, and the dashed line indicate the lack of full knowledge about the mechanisms of iron transport, especially its transendothelial transfer to the fetal blood with the participation of specific polarized ion transporters. Divalent iron ions are marked in green and trivalent iron ions in yellow. ❶—Holo-transferrin (holo-Tf) binds the apical (microvillous) surface of the syncytiotrophoblast to transferrin receptor 1 (TfR1) to form a complex (holo-Tf/TfR1) that undergoes clathrin-mediated endocytosis. ❷—When the pH inside the endosome decreases, Fe^3+^ is released, which is immediately reduced to Fe^2+^ by ferric reductases: the six-transmembrane epithelial antigen of prostate (STEAP) family members, STEAP3 and STEAP4. Divalent iron is released from the endosome into the cytoplasm by divalent metal-ion transporter 1 (DMT1) and ZRT/IRT-like protein 14 (ZIP14), and then, with the participation of poly(rC)-binding protein 1 (PCBP1), a vital intracellular iron chaperone protein, it is stored as ferritin after oxidation to Fe^3+^ in the dinuclear iron-binding ferroxidase center located within the ferritin heavy chain 1 (FTH1) or is exported outside the cell via ferroportin. ❸—Ferrous iron entering the villous core stroma is oxidized to ferric ions by zyklopen (Zp), a multicopper ferroxidase expressed with the basal (fetal-facing) membrane of the syncytiotrophoblast; then, in the form of holo-Tf, it undergoes passive diffusion in the extracellular fluid towards the basolateral surface of endothelial cells, where it binds to TfR1s to form holo-Tf/TfR1 complexes. ❹—The endocytosis of holo-Tf/TfR1 complexes and the storage of iron in the form of ferritin, as well as its transendothelial transport towards fetal capillaries, are essentially similar to the processes occurring in syncytiotrophoblast cells. However, in endothelial cells the dominant representative of the STEAP family is STEAP4, and divalent iron ions released by ferroportin are subject to oxidation by multicopper ferroxidases: hephaestin (HEPH) and ceruloplasmin (CP). ❺—Fetal blood distributes iron-loaded transferrin to rapidly growing tissues that express high levels of TfRs to meet heavy iron demands for cell proliferation, growth, and hemoglobin synthesis.

The main transplacental mechanism of iron transport is shown in Figure 2.

### 5.2. Placental Function in Iron Deficiency

Maternal iron deficiency impairs normal placental development, which limits the effective placental transfer of oxygen and nutrients [177,178]. Serious consequences of maternal IDA include increased maternal mortality, severe postpartum hemorrhage, preterm birth, low birth weight and long-term fetal neurodevelopmental deficits [179,180,181]. Because maternal iron deficiency can result from various causes, each of which can independently affect the placenta and fetus, typical treatment with iron supplements is not always effective [177]. Therefore, a detailed understanding of the molecular mechanisms of placental developmental disorders in individuals with iron deficiency may be crucial for the future development of optimal treatments for anemia during pregnancy [5,182].

#### 5.2.1. Physiological Anemia

Physiological (normal) anemia resulting from the disproportionately greater increase in the plasma volume compared with the increase in red blood cell mass in pregnant women, i.e., the dilution of erythrocytes, usually has a mild course in individuals with hemoglobin levels above 10.5 g/dL. In this case, red blood cell size, measured as the mean corpuscular volume (MCV), remains within the normal range [80 to 100 femtoliters (fL)], which distinguishes this condition from fully developed iron deficiency anemia, which is accompanied by a reduction in MCV (microcytosis) [183]. However, the MCV can remain completely normal during the early phase of iron shortage because stored iron (ferritin) levels decrease before the cell size changes [184,185]. Therefore, major health organizations recommend routine low-dose iron supplementation during pregnancy to support increased nutritional demands and prevent iron deficiency, even if mild hemodilution (physiological anemia) is a normal part of pregnancy [186,187]. One well-described approach to selective supplementation is based on an estimation of iron stores by serum ferritin. A plasma ferritin concentration > 70 µg/L indicates that iron stores are adequate to support pregnancy and that no supplementation is needed, whereas a plasma ferritin concentration less than 30 µg/L may indicate an almost complete lack of iron stores and indicate the need for iron supplementation [188,189].

Reduced blood viscosity caused by physiological hemodilution decreases vascular resistance and facilitates optimal blood flow and increased perfusion within the placenta [190]. Optimal microvascular circulation through the low-pressure intervillous spaces of the placenta supports effective nutrient and gas exchange. Mild decreases in hemoglobin and hematocrit linked to hemodilution may help trigger beneficial vascular growth factor signaling, which is crucial for trophoblast differentiation and placental vessel development [191]. The hypoxic environment in the first trimester of pregnancy plays an important role in hypoxia, which is related to the occlusion of uterine spiral arteries by extravillous trophoblasts (EVTs), resulting in the activation of hypoxia inducible factors (HIFs), which are the main transcription factor regulators that control cellular responses to low oxygen levels [192,193]. Hypoxia-inducible factor 1-alpha (HIF-1α) activation in the developing placenta stimulates angiogenesis by upregulating the expression of angiogenic factors such as vascular endothelial growth factor (VEGF) and enables metabolic adaptation by switching metabolic pathways toward anaerobic processes to reduce oxygen consumption while maintaining energy balance [194,195]. Moreover, the action of HIFs affects almost all stages of trophoblast differentiation and placental growth in both normal and pathological pregnancies, and in addition to low oxygen levels, the secretion of HIFs is influenced by the renin–angiotensin system (RAS) and cytokines and growth factors that regulate placental function and homeostasis [196].

The absence of normal plasma expansion in pregnant women with dilutional anemia may therefore alter healthy microvascular development in the placenta through increased blood viscosity and a lack of modulation of local oxygen tension in placental tissue [177]. Moreover, excessive intake of iron supplements in pregnant women with normal iron stores (ferritin) may increase oxidative stress and potentially disrupt the normal development of small blood vessels in the placenta [15].

#### 5.2.2. Pathological Iron Deficiency Anemia

Iron deficiency during pregnancy, which is associated with a decrease in hemoglobin levels below the values typical for hemodilution and depletion of the intracellular stores of this element, leads to serious disorders of placental function, in which the pathomechanisms of chronic hypoxia play major roles [5,197,198].

Hypoxia itself often exacerbates oxidative stress because of a paradoxical increase in ROS levels [199,200]. Normally, because oxidative metabolism is necessary to meet the needs of the growing fetus, the placenta is a source of ROS. Therefore, in physiological pregnancy, placental antioxidant protective systems function efficiently and neutralize free radicals formed within the uteroplacental–fetal unit [201]. However, a low, physiological level of free radical production is necessary and constitutes an important component of cellular signaling pathways and downstream activities during normal placental development [202]. Only in the case of placental hypoxia, which is often caused by iron deficiency and may be accompanied by microcirculation disorders that limit blood flow or cause fluctuations in blood flow, is the risk of hypoxia–reoxygenation or ischemia–reperfusion injury significantly increased [203,204].

At the mitochondrial level, low oxygen levels slow the activity of the electron transport chain, causing electron carriers to back up and leak single electrons directly to oxygen, resulting in the generation of superoxide [205,206,207]. This occurs because hypoxia triggers enzymes such as nicotinamide adenine dinucleotide phosphate (NADPH) oxidase and xanthine oxidase (XO). NADPH oxidase, a multiprotein enzyme complex, produces ROS by transferring electrons from NADPH to molecular oxygen, whereas xanthine oxidase is converted from xanthine dehydrogenase (XDH) under cellular stress conditions, resulting in the accumulation of purine breakdown products (hypoxanthine and xanthine) [208,209]. While XO cannot fully function without oxygen during the pure hypoxic phase, a critical surge in production occurs upon reoxygenation/reperfusion, where XO uses reintroduced molecular oxygen to produce massive amounts of ROS (mainly hydrogen peroxide and superoxide radicals) [209]. Over time, antioxidant depletion also occurs [210]. Cellular defense systems become less effective and cannot neutralize additional free radicals. Tissue damage occurs, which affects the parenchymal and circulatory (fetal and maternal) compartments. Excessive ROS cause severe damage to cellular lipids (lipid peroxidation), proteins, and DNA, leading to trophoblast dysfunction and cell death [211]. This results in aberrant placentation and immune disturbances, which are linked to many pregnancy-related disorders, including early and recurrent pregnancy loss, fetal death, spontaneous preterm birth, preeclampsia and fetal growth restriction [212,213].

Placental dysfunction is accompanied by morphological changes, which usually represent compensatory but ultimately insufficient adaptations to hypoxia [214]. Placentas from pregnancies complicated by IDA often exhibit hypertrophy with a disproportionately increased weight relative to the birth weight of the fetus. Areas of necrosis (infarcts) and calcifications caused by inadequate blood flow are found in the placental tissue. Deposits of fibrin-like material are present within the intervillous spaces and placental villi, indicating fibrinoid necrosis, which further impairs/blocks nutrient and gas exchange [215]. Chronic villitis occurs with the excess production of syncytial knots, indicating increased trophoblast turnover and apoptosis [216]. While increased capillary proliferation within the villi can be observed in the placenta, endothelial damage and impaired local blood flow regulation can occur because of reduced levels of vascular smooth muscle cells (VSMCs) [215,217].

### 5.3. Iron Overload and Placental Ferroptosis

As a typical micronutrient, iron is characterized by a U-shaped risk curve [218]. Therefore, both iron deficiency and excess iron lead to increased health risks. Iron overload in the human placenta disrupts normal cellular function and is strongly linked to pregnancy complications [219]. Regardless of the many possible causes of placental iron overload, dysregulated transport pathways play major roles in the mechanism of intracellular iron accumulation. Imbalances in the levels of iron-transporting proteins (such as increased TfR1 and DMT1 levels and decreased ferroportin levels) may result in the retention of excess iron within placental trophoblast cells [16]. Moreover, a decrease in the level of intracellular ferritin may reduce the ability of cells to safely store iron, resulting in high levels of reactive free iron, which can result in oxidative damage and cell death [220].

#### 5.3.1. Labile Intracellular Iron and the Fenton Reaction

**Figure 3:**
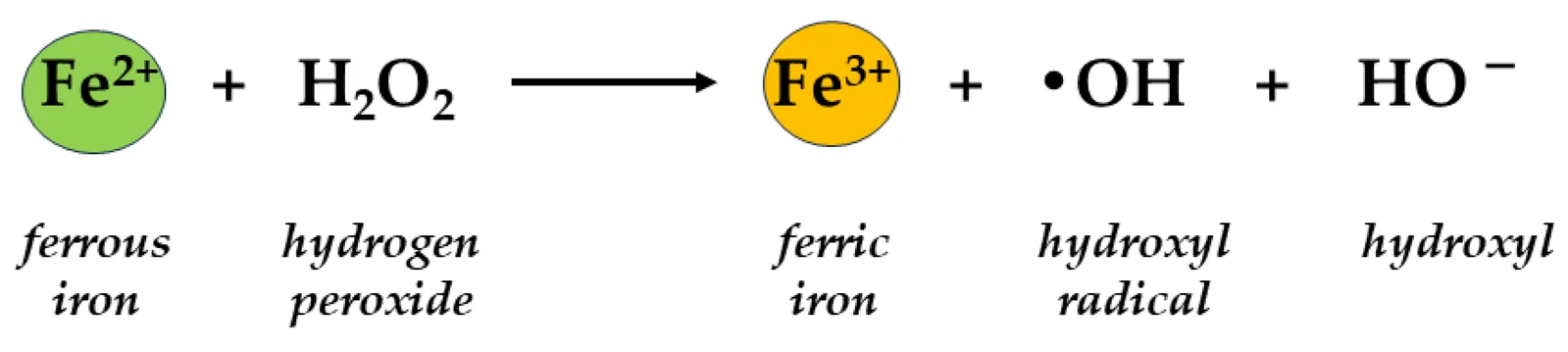
Fenton reaction.

Iron overload has toxic effects, which are primarily a consequence of the presence of excess labile (unbound or loosely bound), i.e., redox-active ions of Fe^2+^ inside the cell, which act as chemical catalysts and transform ordinary cellular byproducts into highly destructive ROS via the Fenton reaction [221]. The **Fenton reaction** is a catalytic process in which ferrous iron (Fe^2+^) reacts with hydrogen peroxide (H_2_O_2_) under acidic conditions to produce highly reactive hydroxyl radicals (•OH) (Figure 3).

Lysosomes and endosomes constitute acidic cell compartments that have a pH of 4.5 to 5.0, which allows iron-mediated radical generation to proceed more efficiently [222]. The hydroxyl radicals generated during the unchecked Fenton reaction fiercely attack nearby biomolecules, causing lipid peroxidation with subsequent ferroptosis (iron-dependent regulated cell death) and protein damage owing to the loss of their normal shape and function [223]. Nucleic acid (DNA/RNA) oxidation leads to damage with an increased risk of genetic mutations (e.g., due to large breaks in nucleic acid strands and the disruption of vital purine bases such as guanine) [224,225].

Moreover, excess labile iron levels via Fenton reactions influence epigenetics by acting as crucial cofactors for iron-dependent modifying eraser enzymes (e.g., ten-eleven translocation (TET) enzymes and Jumonji C (JmjC) domain-containing demethylases), leading to altered DNA and histone demethylation patterns [226,227]. Consequently, excess redox-active Fe^2+^ activity leads to altered chromatin dynamics, with a shift in the balance toward widespread DNA or histone hypomethylation, including reductions in activating marks such as H3K4me3 (trimethylation at the 4th lysine residue of the histone H3 protein) [228]. Thus, as a sensor capable of modulating cAMP signaling pathways, the labile iron pool may reflect and respond to the immediate metabolic demands of cells [229].

In hemochorial placentae, iron is transferred from maternal blood to the fetus through STBs, i.e., the outer lining of the placenta that contacts maternal blood; these cells show very high activity in iron metabolism and may be susceptible to excessive iron accumulation under certain conditions [16,230]. Moreover, recent reports have provided evidence that in states of severe iron deficiency during pregnancy, the placenta begins to prioritize its own metabolic needs and, as a compensatory reaction, may excessively store iron instead of—as previously thought—unconditionally eliminating ferritin and providing iron to the fetus [23,157,173].

#### 5.3.2. Ferroptosis in Placental Tissue

Ferroptosis: An overview.

Ferroptosis was discovered and named in 2012 by scientist Dr. Scott W. Dixon and principal investigator Dr. Brent R. Stockwell alongside their research team at Columbia University [231]. They identified it as a unique form of regulated, iron-dependent regulated cell death driven by the unchecked accumulation of lipid peroxides. Although some types of autophagy (e.g., ferrinophagy), a process that usually keeps cells alive by degrading, clean up, and recycling their own damaged or unnecessary cellular components, can actually help trigger ferroptosis, the two processes should be treated separately [232]. Ferroptosis differs significantly from apoptosis, which is dominated by cell shrinkage and caspase activation, and from necrosis, which is characterized by uncontrolled swelling and bursting [233]. In contrast, iron-dependent cell death is defined by specific physical and biochemical traits [234,235,236,237].

**The physical and morphological traits of ferroptosis include** mitochondrial alterations with reduced volumes (mitochondrial shrinkage), increased membrane density, elevated membrane potentials, reductions in the levels of or total disappearance of inner mitochondrial cristae, and outer mitochondrial membrane rupture alongside intact plasma or cellular membrane breakdown at later stages [238,239]. A characteristic feature of ferroptosis is the preservation of nuclear integrity, i.e., no chromatin condensation or DNA fragmentation occurs, as observed in apoptosis [240,241].

**The biochemical traits of ferroptosis include the following**:

Iron overload, where the accumulation of labile, redox-active intracellular iron triggers the generation of ROS via the Fenton reaction [50];

Lipid peroxidation associated with the destructive accumulation of oxidized polyunsaturated fatty acids (PUFAs) in cell membranes [242];

Antioxidant failure, including the depletion of glutathione (GSH) and inactivation of the lipid repair enzyme glutathione peroxidase 4 (GPX4), as well as decreased activity of ferroptosis suppressor protein 1 (FSP1) [243,244];

Loss of key regulatory metabolic drivers and inhibition of membrane transporters that provide vital antioxidant building blocks (e.g., system Xc–, which is the functional cystine–glutamate antiporter), as well as increased activity of cell-surface iron transporters (such as transferrin) [245,246].

**Figure 4:**
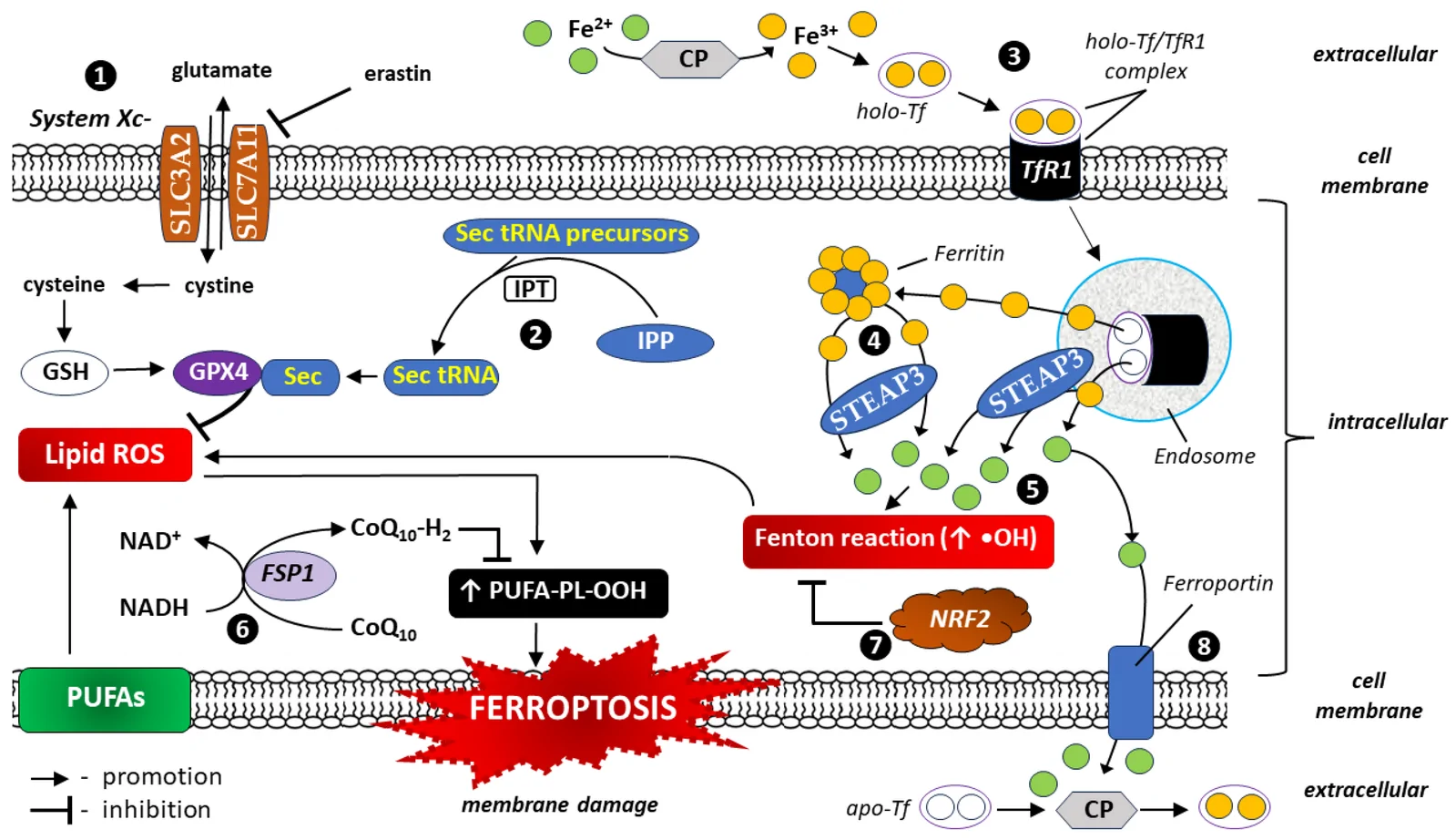
Key regulators and major signaling pathways of ferroptosis [50,235,237,245,247]. ❶—The system Xc- (cysteine/glutamate antiporter), an amino acid transporter consisting of two specific protein subunits: the solute carrier family 7 member 11 (SLC7A11) that handles the exchange of glutamate with cystine and a heavy chain subunit, a solute carrier family 3 member 2 (SLC3A2) required for proper cell-surface trafficking and stabilization. The exchange of glutamate with cystine via the Xc- system is required to import cystine into cells, which, after reduction to active cysteine inside the cytoplasm, serves for the synthesis of the major cellular antioxidant glutathione (GSH). Glutathione peroxidase 4 (GPX4) is a crucial antioxidant enzyme that regulates ferroptosis by turning harmful lipid reactive oxygen species (lipid ROS) into their corresponding non-toxic lipid alcohols. Erastin can block the uptake of cystine mediated by SLC7A11 and induce cell death by ferroptosis. ❷—Proper activity of the mevalonate pathway is essential for selenocysteine (Sec) utilization and synthesis by GPX4. This metabolic route produces isopentenyl pyrophosphate (IPP), which is critical for modifying and maturing selenocysteine tRNA. Then, under the influence of isopentenyltransferase (IPT), Sec is incorporated into proteins, including GPX4. IPP deficiency causes an inability to properly incorporate Sec, leading to GPX4 depletion and high susceptibility to lipid peroxidation and ferroptosis. ❸—The intracellular transfer of iron mediated by transferrin receptors (TfR1) is a critical driver of ferroptosis. By binding and internalizing extracellular transferrin-bound iron, TfR1 continually feeds the cytoplasmic labile iron pool. Excessive iron accumulation promotes lethal lipid peroxidation through Fenton reactions, making TfR1 expression a key checkpoint in cell susceptibility to ferroptosis. ❹—The six-transmembrane epithelial antigen of prostate (STEAP) proteins, especially STEAP3, reduce ferric iron (Fe^3+^) to ferrous iron (Fe^2+^). Therefore, STEAP activity may be, together with ferritin storage level, crucial for labile iron pool (❺) management in the context of ferroptosis. ❻—Ferroptosis suppressor protein 1 (FSP1) blocks ferroptosis by acting as a nicotinamide adenine dinucleotide NAD(P)H–coenzyme (CoQ_10_) oxidoreductase to promote production of ubiquinol (CoQ_10_-H_2_). Ubiquinol, as a powerful lipophilic antioxidant, prevents excessive transformation of polyunsaturated fatty acids (PUFAs) into polyunsaturated fatty acid-containing phospholipid hydroperoxide (PUFA-PL-OOH) under the influence of lipid ROS. ❼—Nuclear factor erythroid 2-related factor 2 (NRF2) acts as a crucial negative regulator that limits the damaging effects of hydroxyl radicals (•OH) during the Fenton reaction. NRF2 acts as a master transcription factor against ferroptosis by controlling key genes involved in antioxidant defense (e.g., *SLC7A11*), iron handling/storage, and lipid repair. ❽—Normal ferroportin activity protects cells from ferroptosis by exporting excess intracellular iron and lowering the cytoplasmic labile iron pool prevents the Fenton reactions. Conversely, low ferroportin activity causes intracellular iron accumulation, promotes oxidative stress, and accelerates cell death via ferroptosis. CP—ceruloplasmin.

Two mechanisms are involved in ferroptosis: the intrinsic mechanism, which is an enzyme-regulated cell death pathway driven by the iron-dependent accumulation of lethal lipid peroxides within cell membranes due to failing internal antioxidant defense mechanisms, and the extrinsic (transporter-dependent) mechanism, which is triggered by extracellular signals and the regulation or inhibition of specific cell membrane transporters that control amino acid exchange and iron uptake [247]. The main signaling pathways involved in ferroptosis are summarized in Figure 4.

Importantly, a **definitive demonstration of ferroptosis** requires a multi-parametric experimental validation combining genetic, biochemical, and morphological evidence, rather than relying on a single marker. Because many molecular changes seen in ferroptosis also occur in other forms of cell death or cellular stress, specific criteria must be met to definitively claim that ferroptosis has occurred. In the field of cell death research, distinguishing between ferroptosis-associated molecular alterations and the definitive demonstration of ferroptosis is the difference between identifying potential clues or indicators (e.g., lipid peroxidation accumulation, antioxidant system depletion, iron overload signaling and morphological alterations) and proving a cause-of-death verdict. Definitive demonstration of ferroptosis requires functional proof that the cell death process is mechanistically driven by iron-dependent lipid peroxidation and can be actively halted by specific, targeted inhibitors. According to guidelines established by the Nomenclature Committee on Cell Death (NCCD), a definitive demonstration requires the following evidence [248]:

Pharmacological rescue (the gold standard), when the cell death must be prevented, delayed, or significantly mitigated by treatment with highly specific ferroptosis inhibitors (e.g., lipid trapping antioxidants, iron chelators). If these agents do not save the cells, the death is not ferroptosis.

Genetic or pharmacological sensitization, where manipulation of specific enzymes responsible for fueling or preventing ferroptosis must predictably alter the death rate.

Exclusion of other death pathways, when blocking other major regulated cell death pathways (e.g., apoptosis, necroptosis) does not prevent the cell death.

Following the criteria for definitively demonstrating ferroptosis can undermine the results of most recent studies, which usually identify potential indicators of ferroptosis.

#### 5.3.3. Placental Ferroptosis and Pregnancy Complications

The subtle shift from normal placental iron homeostasis to ferroptosis.

Maintaining iron homeostasis in trophoblast cells and endotheliocytes of the villous microcirculation is a challenge at every stage of pregnancy [22,146,249]. The delicate threshold between normal placental iron metabolism and ferroptosis depends on the balance among iron uptake, storage, and antioxidant defense mechanisms [19]. Under normal conditions, the placenta tightly regulates iron uptake via transferrin receptors, controls its intracellular reserve pool via ferritin stores, and exports iron via ferroportin to nourish the fetus while preventing toxicity [157]. When this threshold is crossed, iron overload in trophoblasts triggers destructive, iron-dependent lipid peroxidation that compromises placental function and drives severe obstetrical complications [19,157].

Under physiological conditions, excess iron typically results in the downregulation of TfR1; however, pathological placental overexpression of TfR1, which is found in preeclampsia, accelerates iron accumulation, triggering oxidative stress and cellular ferroptosis [13,16].

An example of the narrow boundary between the control of cellular iron cycling and the occurrence of programmed iron-dependent cell death is ferritinophagy [250]. During this process of selective autophagy, the receptor protein nuclear receptor coactivator 4 (NCOA4, also known as androgen receptor activator 70 (ARA70)) binds to the heavy chain 1 subunit of ferritin (FTH1) within the iron-storage ferritin complex [251]. The NCOA4–ferritin complex is subsequently transported toward and engulfed by autophagosomes and ultimately delivered to lysosomes for degradation [252]. After such ferritin decays, trivalent iron ions are reduced to divalent iron (ferrous Fe^2+^) and almost simultaneously released into the cytosol, which directly increases the intracellular labile iron pool to meet cellular iron demands [253]. The replenishment of or an increase in the labile iron pool is a necessary compensatory mechanism (to adjust demand) for intensive maternal–fetal iron transfer, and the autophagic pathway is utilized to mobilize iron from intracellular stores [254]. Expanding the cytosolic iron pool ensures that the developing placenta and fetus receive adequate amounts of iron even when maternal supply fluctuates. However, unchecked ferritinophagy can result in the overexpansion of the labile iron pool, which is located primarily in the cytosol but is also present in other cellular compartments, such as mitochondria, the nucleus and lysosomes [255]. Under such conditions, excess weakly bound, redox-active iron has toxic effects, generating ROS and triggering iron-dependent cell death (ferroptosis) [256,257].

The coexistence of oxidative stress, during which excess ferrous iron (Fe^2+^) reacts with hydrogen peroxide via the Fenton reaction, resulting in the generation of massive amounts of ROS, and antioxidant collapse, which prevents the neutralization of lipid hydroperoxides due to a critical reduction in the levels of or failure of protective enzymes—most notably GPX4—almost always indicates the occurrence of placental ferroptosis [18,258].

Iron homeostasis may also shift toward ferroptosis in the placenta owing to decreased activity or downregulation of the activity of ferroportin, the primary cellular iron exporter [259]. The iron trapped intracellularly in trophoblasts and endothelial cells causes toxic lipid peroxidation and the generation of ROS, which directly drives ferroptosis and contributes to placental dysfunction in several pregnancy complications [16,18,260].

Ferroptosis is associated with pregnancy complications.

In recent years, increasing evidence has shown that an iron-dependent form of regulated cell death driven by lipid peroxidation may contribute to placental insufficiency and related pregnancy complications. Ferroptosis plays an important role in the pathophysiology of placental abnormalities associated with often overlapping pregnancy complications, such as preeclampsia, gestational diabetes mellitus, fetal growth restriction, spontaneous abortion (miscarriage), and preterm birth [260].

In **preeclampsia**, a high blood pressure disorder that typically develops after the 20th week of pregnancy with symptoms of multiorgan damage (e.g., liver, kidneys, and central nervous system), iron dyshomeostasis with iron overload and subsequent ferroptosis contributes to placental damage, especially within the STB [261]. The significant contribution of iron metabolism disorders to the still insufficiently understood pathomechanisms of preeclampsia is suggested by elevated hemoglobin levels, increased transferrin saturation (↑ holo-Tf), elevated ferritin and serum iron levels during the second trimester, and placental iron overload [262,263]. Analyses of iron transporters by single-cell/nuclear RNA sequencing revealed increased expression of the TFRC and SLC11A2 genes, which encode TfR1 and DMT1, respectively, and reduced expression of *SLC40A1*, which encodes ferroportin [16]. The above changes, indicating iron overload, were accompanied by typical signs confirming increased ferroptosis in trophoblast cells, such as increased ROS levels and increased levels of 4-hydroxy-2-nonenal (4-HNE), a toxic, reactive aldehyde produced as a major end product during the lipid peroxidation of PUFAs, as well as reduced expression of the ferroptosis inhibitor GPX4 [264]. The above symptoms related to oxidative stress manifested as reduced placental secretion of progesterone (P4) and placental growth factor (PlGF) [264]. Preeclampsia involves a systemic hyperinflammatory state and immune dysregulation, which amplify endothelial damage and placental dysfunction [265,266]. Elevated levels of proinflammatory cytokines such as interleukin-6 (IL-6) stimulate the liver and placenta to produce more hepcidin, which targets ferroportin and regulates iron metabolism by preventing the release of iron into the bloodstream, trapping it inside cells and promoting its storage [267,268]. Some researchers have proposed that high hepcidin expression in preeclampsia acts as a protective response (compensatory mechanism) to limit free iron levels, oxidative stress, and endothelial damage [268,269,270]. Elevated serum hepcidin levels during early pregnancy are associated with preeclampsia [270]. However, meta-analyses suggest elevated serum hepcidin levels in preeclampsia, a definitive clinical consensus has not yet been reached because conflicting individual studies suggest no significant correlation among iron status, hepcidin levels and IL-6 levels or even question the increase in hepcidin levels in preeclampsia [271,272]. In the latter case, the authors concluded that high serum iron levels in preeclamptic women are likely caused by low hepcidin production [272].

The inconsistent correlations observed in preeclampsia research, including those related to hepcidin and iron metabolism, may be related to the heterogeneous nature of the condition, which has diverse underlying causes and clinical presentations [273].

Genetic studies have shown that in preeclampsia, reduced expression of the FTH1 (ferritin heavy chain 1) and FTL (ferritin light chain) genes, which encode the respective subunits of ferritin, may be associated with disturbances in the safe intracellular storage of iron in the form of Fe^3+^ and an increase in the labile pool of Fe^2+^ involved in the Fenton reaction [274,275]. For example, specific microRNAs (such as miR-150-3p) are upregulated in preeclampsia and directly target and downregulate FTH1 expression [275].

In preeclampsia, trophoblast ferroptosis may occur owing to the influence of the microRNA miR-30b-5p, which may simultaneously suppress the antiporter cystine/glutamate (system Xc–) and transcription factor paired box gene 3 (PAX3) [276]. This suppression promotes ferroptosis by decreasing the level of GSH and increasing the concentration of Fe^2+^ in the labile pool. In turn, the abnormally upregulated expression of miR-210 in preeclampsia leads to iron accumulation and autophagosome formation in trophoblast cells and hemosiderin deposition in placental stroma trophoblasts [277].

In terms of the link between the pathomechanism of preeclampsia and iron metabolism, the influence of iron supplementation in iron-deficient women, which may inhibit maternal hepcidin production and function, remains unclear. Absorption of dietary iron may increase, which may subsequently lead to an increase in hemoglobin levels, an increase in blood viscosity, and ultimately a reduction in placental blood flow (placental ischemia), which predisposes patients to endothelial dysfunction and the release of factors that increase blood pressure due to vasoconstriction (e.g., endothelin 1) [51]. Moreover, excessive dietary iron intake increases the risk of increased NTBI content in blood plasma, which is transported through the placenta and causes placental dysfunction because of increased oxidative stress, lipid peroxidation, and DNA damage [22].

In **gestational diabetes mellitus (GDM)**, a common metabolic complication of pregnancy associated with adverse maternal and fetal outcomes, ferroptosis contributes to the development of placental dysfunction through an interconnected network involving oxidative stress, inflammation and impaired angiogenesis [278,279]. Placental tissue studies in GDM patients revealed elevated levels of oxidative stress markers, including phagocyte NADPH oxidase 22-kilodalton subunit (P22PHOX) and thioredoxin-interacting protein (TXNIP), and decreased levels of antioxidant markers such as heme oxygenase 1 (HO-1), catalase (CAT), superoxide dismutase 2 (SOD2) and NAD(P)H quinone dehydrogenase 1 (NQO1) [221]. Consistently, the levels of the anti-ferroptotic markers GPX4, SLC7A11, and NRF2 were significantly decreased, whereas the levels of the pro-ferroptotic markers TfR1 and acyl-CoA synthetase long-chain family member 4 (ACSL4) were increased, suggesting increased iron accumulation and lipid peroxidation [258,280]. The development of a chronic inflammatory background in diabetic placental tissue was confirmed by increased levels of proinflammatory cytokines (IL-6, interleukin-1β (IL-1β), and tumor necrosis factor alpha (TNF-α)) and the activation of nuclear factor kappa-light-chain-enhancer of activated B cells (NF-κB), as well as the reduced expression of interleukin-10 (IL-10), which is primarily an anti-inflammatory cytokine [281,282]. Recent studies have shown that placental hepcidin mRNA and protein levels are significantly increased in diabetic pregnancies compared with those in healthy controls [18]. This upregulation of hepcidin expression is likely driven by the inflammatory response in diabetic placental tissue. Increased hepcidin expression occurs predominantly in the STB layer of the placenta and is correlated with downregulated ferroportin expression. The expression of hepcidin in individuals with GDM may also be influenced by polymorphisms of the hepcidin gene, in addition to iron nutritional status and the degree of compensation for glycemic disorders [283]. Ferritin translation and iron storage in the placenta are often suppressed in complicated or poorly controlled diabetic pregnancies, particularly when accompanied by fetal iron deficiency [284]. The expression of TfR1 varies depending on the specific type of diabetes, local iron status, and the presence of oxidative stress or hyperglycemia. Studies have reported conflicting patterns; some studies have reported increased TfR1 expression or mRNA accumulation in diabetic pregnancies complicated by low fetal iron stores, whereas other recent studies on GDM and diabetes in pregnancy (DIP) have reported decreases or no significant changes in TfR1 protein levels in the STB [18,285,286]. It is assumed that, especially in poorly controlled diabetes during pregnancy, hyperglycemia and lipid levels alter iron handling pathways and trigger oxidative stress, which can decrease TFR1 expression and the expression of other iron transporters, such as DMT1 and FPN1, as a protective cellular response [287].

Ferroptosis also acts as a central driver of impaired placental angiogenesis and dysfunction in GDM [258,288]. The combined burden of oxidative stress and ferroptosis suppresses vital vascular markers (key vascular development regulators), such as vascular endothelial growth factor A (VEGF-A), hypoxia-inducible factor (HIF) 1 α subunit (HIF-1α) and stromal cell–derived factor 1 (SDF-1α, also known as chemokine CXCL12), thereby stunting healthy blood vessel growth in the placenta [280]. Angiogenesis disorders in the diabetic placenta are characterized mainly by excessive, abnormal branching of vessels (hypervascularization) and disturbances in their maturation and structure [289].

Table 2 is an attempt to summarize and organize the knowledge on the involvement of ferroptosis in preeclampsia and GDM.

Emerging obstetric research has confirmed that ferroptosis may contribute to **fetal growth restriction, spontaneous abortion**, and **preterm birth** by triggering placental trophoblast injury and disrupting maternal–fetal tolerance [260]. Excessive cell death at the maternal–fetal interface compromises syncytialization, nutrient and gas transport, and placental hormone secretion, compromising proper growth and development of the fetus at various stages of pregnancy [264]. Disturbed iron metabolism and severe oxidative stress, which overwhelm placental antioxidant defense mechanisms and drive extensive ferroptotic cell death in trophoblasts, are responsible for the following processes:

Impaired early embryonic tissue invasion and breakdown in the maternal–fetal immune tolerance necessary to sustain pregnancy [290,291];

Fetal growth restriction in the subsequent weeks of pregnancy due to limited angiogenesis and blood vessel remodeling, causing tissue underperfusion and hypoxia–reoxygenation cycles, which lead to the acceleration and accumulation of lipid ROS [257,292];

Preterm birth, as ferroptosis prematurely damages the integrity of placental membranes and chorionic tissues and leads to hormonal disruptions in the placenta, which may trigger signaling cascades that stimulate premature uterine contractile activity [19,293].

Recommended remedies and potential therapeutic options to prevent placental ferroptosis-related complications.

General recommendations, although not yet formulated into clinical practice guidelines, may include avoiding cellular iron overload and excessive lipid peroxidation.

**Iron supplementation during pregnancy should be monitored**, preferably under medical supervision, to avoid unnecessary administration/taking an excessive dose. Treatment with iron preparations should be initiated after a clinical diagnosis of IDA because excess circulating non-transferrin-bound iron promotes placental oxidative damage (ferroptosis) [258,294].

Any **inflammation** during pregnancy should be treated effectively, considering the level of oxidative stress associated with inflammation [219,257].

The **BMI should be normalized**, as larger BMI values indicating obesity (BMI ≥ 30) are associated with the development of chronic low-grade inflammation (CLGI or metainflammation). To this end, obesogenic dietary habits (avoiding high-fat/high-sugar diets) that disrupt maternal–placental iron handling and increase the expression of markers of iron-dependent lipid damage should be minimized [295,296].

Pregnant women should especially avoid **environmental toxins** such as cigarette smoke (cessation of smoking) and exposure to environments with poor air quality (smog) or the risk of exposure to substances classified as endocrine-disrupting chemicals (EDCs) such as bisphenol A (BPA) [292,297,298].

**Antioxidant defenses** should be supported by maintaining adequate dietary intake of natural antioxidants (e.g., green tea and resveratrol from grapes) and nutrients that support GSH and cellular defense pathways [299,300].

**Pregnancy-related conditions**, especially blood pressure and metabolic homeostasis (especially glucose control), should be closely managed because hypertension (preeclampsia) and GDM actively increase susceptibility to placental ferroptosis [260,279,301].

**Vitamins with antiferroptotic effects** include water-soluble vitamin B6 (pyridoxine), which helps restore the proper level of glutathione; fat-soluble vitamin E (tocopherol) and vitamin K, which inhibit lipid peroxidation directly in the cell wall; and vitamin A (retinol) and vitamin D (calciferol), which activate genes that protect cells against ferroptosis [302,303].

The above recommendations show that following the general principles of proper pregnancy management, including nutrition, significantly reduces the risk of placental ferroptosis. However, with regard to monitoring iron supplementation for the prevention of ferroptosis, there are currently no official guidelines for use in clinical practice. Standard antenatal care focuses instead on preventing and treating maternal IDA. Similarly, there are currently no official clinical guidelines or standard recommendations for using “anti-ferroptotic” vitamin treatments during pregnancy.

The pharmacological inhibition of ferroptosis remains in the preclinical development phase and involves three main groups of compounds:

**Iron chelators** that bind tightly to excess iron to safely remove trivalent (e.g., deferoxamine) or divalent (e.g., alpha-lipoic acid) ions outside the cell [304,305];

**Ferroptosis inhibitors** or radical-trapping agents, such as liproxstatin-1 and ferrostatin-1, that act as free radical scavengers and capture ROS before they cause lipid peroxidation [306,307,308];

**Repurposed drugs**, including dipyridamole and promethazine, in new therapeutic applications such as lipid peroxidation inhibitors, GSH preservation agents, and inhibitors of harmful antiangiogenic factor release [e.g., soluble fms-like tyrosine kinase-1 (sFlt-1 or sVEGFR-1)] in preeclamptic placental tissue [309,310].

Once again, none of the above pharmacological treatments have yet been included in official clinical guidelines or standard recommendations for the treatment of ferroptosis.

The effectiveness of future therapeutic approaches in limiting the harmful effects of ferroptosis in the placenta depends on the ability to locally enhance antioxidant pathways, e.g., by activating GPX4, replenishing GSH (e.g., supplying precursors or increasing cellular pools of GSH) and targeting protective enzymes [e.g., calcium-independent phospholipase A2 group VI (PLA2G6)] that repair peroxidized phospholipids in placental cells [311,312]. Considering that nuclear factor erythroid 2-related factor 2 (NRF2) regulates genes responsible for antioxidant and detoxification responses, activation of the p62 (sequestosome 1, SQSTM1)-kelch-like ECH-associated protein 1 (KEAP1)–NRF2 signaling axis in trophoblasts may support the maintenance of redox homeostasis and limit the severity of the Fenton reaction [313,314]. Restricting excessive iron uptake (such as by targeting high TfR1 expression) or preventing NCOA4-mediated ferritinophagy could also prevent dangerous local iron overload during hypoxia in early pregnancy [251,315].

## 6. Concluding Remarks

Achieving and maintaining iron homeostasis during pregnancy is a major challenge for the regulatory systems of pregnant women. The dynamics of iron metabolism during fetal development are particularly complex, and the placenta, as an intermediary organ between the mother and the fetus, plays a central role. Syncytiotrophoblast (STB) cells are specialized placental cells that efficiently absorb, store, and transport iron within the uteroplacental–fetal unit. In a normal pregnancy, maternal iron stores—measured as serum ferritin levels—frequently decrease because of the increase in the plasma volume and high fetal–placental demands, whereas the placental STB, i.e., the outer epithelial layer of the hemochorial placenta, adapts by upregulating TfR1s expression to maximize iron transfer to the fetus. Owing to the higher receptor density, the STB can effectively capture deficient iron from maternal circulation and quickly transport it via the prominently expressed ferroportin, an iron scavenger. This decrease in placental iron content reflects normal iron utilization rather than an immediate pathological state, although severe decreases may result in iron deficiency. The lack of iron accumulation and efficient antioxidant defense mechanisms indicate that the labile intracellular ferrous iron (Fe^2+^) pool does not pose a significant risk for ferroptosis, a form of regulated cell death driven by iron-dependent lipid peroxidation and the unchecked accumulation of ROS in cell membranes. However, in cases of placental iron overload (e.g., due to unmonitored excessive iron supplementation) and pathological pregnancy with concomitant oxidative stress, an increased pool of toxic Fe^2+^ combined with high oxygen fluctuations (such as early placental hypoxia/reperfusion) can trigger Fenton reactions and subsequent lipid peroxidation, leading to ferroptosis-related placental dysfunction. Placental dysfunction due to lipid peroxidation often develops in the context of chronic inflammation, which exhausts the antioxidant defense capacity. This transition from physiology to pathophysiology in the placenta is often difficult to capture in clinical studies. CLGI is closely linked to obesity during pregnancy, GDM and hypertensive disorders such as preeclampsia, which frequently co-occur as a pregnancy-specific metabolic syndrome. Therefore, considering the global epidemic of metabolic disorders in pregnant women, targeting placental ferroptosis could help in the prevention and treatment of serious pregnancy complications, thereby improving obstetric outcomes. However, despite the well-known health-promoting recommendations for pregnant women, including maintaining a healthy body weight and optimal dietary composition, including supplementation of iron, vitamins and natural antioxidants, research on the pharmacological inhibition of ferroptosis has not progressed beyond the preclinical phase.

## Figures and Tables

**Table 1:** Iron compartments in the human body [3,32,42,51]. DMT1—divalent metal-ion transporter 1.

Main Pool	Compartments Within the Main Pool	Iron Content (mg)	Percentage of the Total Iron	Key Associated Protein(s)	Main Physiological Role
Functional pool	Erythrocytes and erythroid precursors	1650–3000 mg	50–60%	Hemoglobin	Binding and transport of oxygen from the lungs to the tissues.
	Muscle cells	300–500 mg	~10%	Myoglobin	Intracellular oxygen store and carrier in myocytes.
	Cellular enzymes (enzymatic pool)	150–250 mg	~5%	Cytochromes, catalases	Cellular respiration and ATP energy production (cytochromes); cell protection against oxidative stress (catalases).
Storage pool	Liver parenchymal cells	650–1250 mg	20–25%	Ferritin, hemosiderin	Maintaining iron homeostasis by ensuring mobilizable cellular iron reserves.
	Reticuloendothelial macrophages (spleen, liver and bone marrow)	200–500 mg	5–10%	Ferritin, hemosiderin	Recycling iron through erythrophagocytosis.
Transport (transit) pool	Blood plasma	3–4 mg	≤0.1%	Transferrin (bloodstream), ferroportin (transmembrane) and DMT1 (intracellular)	Dynamic iron transfer between sites of absorption and utilization.

**Table 2:** Iron homeostasis disorders and the proposed involvement of ferroptosis in the pathomechanisms of preeclampsia and GDM based on studies of ferroptosis-related markers in the placenta.

Pregnancy Complication	Study [Ref.]/Model	Human (H)/Animal (A)/In Vitro (IV) Evidence	Ferroptosis-Related Markers	Iron Homeostasis Alterations	Proposed Mechanism	Main Limitations
Preeclampsia (PE)	[16]/Integrated clinical data, a reduced uterine perfusion pressure mouse model, in vitro cell experiments and placental organoids derived from patients with PE	H + A + IV	4-hydroxy-2-nonenal (4-HNE) and reactive oxygen species (ROS) levels, expression of the ferroptosis inhibitor glutathione peroxidase 4 (GPX4)	Elevated Hb levels, increased transferrin saturation (↑ holo-Tf), elevated ferritin and serum iron levels during the second trimester with placental iron overload.	Increased expression of the iron transporters genes TFRC and SLC11A2 genes, which encode TfR1 and DMT1, reduced expression of *SLC40A1*, which encodes ferroportin	Genetic, inflammatory, and environmental factors triggering placental iron accumulation have not been studied. Limitations in fully addressing accurate maternal iron intake and the use of unprescribed iron supplements despite the diagnosis of systemic and placental iron overload in PE patients
	[276]/Human PE placental sample analysis and a reduced uterine perfusion pressure rat model	IV (human) + A	Glutathione (GSH) content and GPX activity, malondialdehyde (MDA), miR-30b-5p expression, total and labile Fe (Fe^2+^) contents, cell viability, lactate dehydrogenase (LDH), intracellular ROS	Intracellular iron accumulation due to decreased iron export, increased labile ferrous iron within the cytoplasm.	Upregulation of miR-30b-5p in PE models plays a pivotal role in trophoblast ferroptosis under hypoxic conditions, by downregulating Cys2/glutamate antiporter (SLC7A11) and paired box 3 (PAX3) and decreasing ferroportin 1 expression, resulting in decreased glutathione (GSH) and increased labile Fe^2+^.	Full translation of data from studies on a homogeneous animal model of PE (reduced uterine perfusion pressure by aortic clipping) with the results of assays on human placental samples is not possible, also due to the clinical and mechanistic heterogeneity of PE.
Gestational diabetes mellitus (GDM)	[18]/Cross-sectional analytical study	H	Placental expressions of hepcidin, ferritin, ferroportin 1, TfR1, TGF-β, NAD(P)H:quinone oxidoreductase 1 (NQO1) and GPX4.	Profound disruption of placental iron homeostasis by increased expression of hepcidin, accompanied by reduction in TfR1, ferroportin 1 and GPX4	Hyperglycaemia and inflammation in GDM can upregulate hepcidin expression, which together with suppression of key iron transport proteins and reduced GPX4, disrupt placental iron transport, promoting intracellular iron retention and ferroptosis in a pro-oxidative placental environment.	Lack of validation of β-actin expression, used as a housekeeping gene in this study, in hypoxia, oxidative stress and metabolic disorders. The study’s findings need to be replicated in populations with a higher prevalence of obesity. Sex-specific differences were not taken into account, although the fetal sex may affect placental mitochondrial function and oxidative stress response. The influence of insulin exposure on placental molecular findings was not fully evaluated.
	[258]/Human GDM placental sample analysis	IV (human)	Placental expression of heme oxygenase 1 (HO-1), NQO1, superoxide dismutase 2 (SOD2), catalase (CAT), GPX4, SLC7A11, nuclear factor erythroid 2-related factor (NRF2), TfR1, acyl-CoA synthetase long-chain family member 4 (ACSL4)	Enhanced iron accumulation and lipid peroxidation in placental tissue co-occurring with increased expression of pro-ferroptotic markers TfR1 and ACSL4.	Hyperglycemia in GDM disturbs placental iron homeostasis, resulting in increased iron overload and inhibition of NRF2 activation, which creates a pro-ferroptotic environment with reduced levels of GPX4 and SLC7A11, as well as increased levels of ACSL4. This results in elevated placental inflamma-tion by NF-κB activation and enhanced pro-inflammatory cytokines, intensifying oxidative stress.	Studies of changes in ferroptosis-associated markers were performed at the mRNA and protein levels, without direct biochemical studies of ferroptosis (e.g., MDA, GSX). Therefore, the results provide indirect evidence for ferroptosis and should be interpreted as associative rather than causal. Maternal BMI was significantly higher in GDM patients compared to the control group, which could be responsible for the increased oxidative stress in placental tissue.

## Data Availability

No new data were created or analyzed in this study. Data sharing is not applicable to this article.

## Abbreviations

4-HNE	4-hydroxy-2-nonenal (aldehyde)
ACSL4	acyl-CoA synthetase long-chain family member 4
ARA70	androgen receptor activator 70, also known as nuclear receptor coactivator 4 (NCOA4)
ATP	adenosine 5′-triphosphate
BMI	body mass index
BMP	bone morphogenetic protein
BMP6	bone morphogenetic protein 6
BPA	bisphenol A
cAMP	cyclic adenosine monophosphate
CAT	catalase
CLGI	chronic low-grade inflammation, also known as metainflammation
CoQ_10_-H_2_	ubiquinol
CP	ceruloplasmin
DcytB	enzyme duodenal cytochrome b
DIP	diabetes in pregnancy
DMT1	divalent metal-ion transporter 1
ECs	endothelial cells (ECs)
EDCs	endocrine-disrupting chemicals
EPO	erythropoietin
ER^+^	estrogen receptor-positive (cells)
EREs	estrogen response elements
ERFE	erythroferrone
EVTs	extravillous trophoblasts
ERFE	erythroid-derived hepcidin-suppressing factor erythroferrone
FLVCR1	feline leukemia virus subgroup C receptor 1
FSP1	ferroptosis suppressor protein 1
FTH1	heavy chain 1 subunit of ferritin
FTL	ferritin light chain
FXIa	factor Xia, also known as activated plasma thromboplastin antecedent or Rosenthal factor
GDM	gestational diabetes mellitus
GPR30	G-protein-coupled estrogen receptor 1
GPX4	glutathione peroxidase 4
GSH	glutathione
H_2_O_2_	hydrogen peroxide
H3K4me3	tri-methylation at the 4th lysine residue of the histone H3 protein
HCP1	heme carrier protein 1, also known as proton-coupled folate transporter (PCFT)
HDs	hemosiderin deposits
HEPH	hephaestin
HFE	homeostatic iron regulator (gene)
HIF-1α, HIF-2α	hypoxia-inducible factors 1-alpha and 2-alpha
HIFs	hypoxia inducible factors
HO-1, HO-2	heme oxygenases 1 and 2
HREs	hypoxia-response elements
IDA	iron deficiency anemia
IL-1β	interleukin 1-beta
IL-6	interleukin-6
IL-10	interleukin-10
IPP	isopentenyl pyrophosphate
IPT	isopentenyltransferase
IREs	iron-responsive elements
IRP1, IRP2	iron regulatory proteins 1 and 2
KEAP1	kelch-like ECH-associated protein 1
LDL	low-density lipoprotein
LRP1	low-density lipoprotein (LDL) receptor-related protein 1
LSECs	liver sinusoidal endothelial cells
MCV	mean corpuscular volume
MPS	mononuclear phagocyte system
NADPH	nicotinamide adenine dinucleotide phosphate
NCOA4	nuclear receptor coactivator 4, also known as androgen receptor activator 70 (ARA70)
NF-κB	nuclear factor kappa-light-chain-enhancer of activated B cells
NQO1	NAD(P)H quinone dehydrogenase 1
NRAMP1	natural resistance associated macrophage protein 1
NRF2	nuclear factor erythroid 2-related factor 2
NTBI	non-transferrin bound iron
P22PHOX	phagocyte NADPH oxidase 22-kilodalton subunit
P4	progesterone
PAX3	paired box gene 3 (transcription factor)
PCBP1	poly(rC)-binding protein 1
PCFT	proton-coupled folate transporter, also known as heme carrier protein 1 (HCP1)
PLA2G6	calcium-independent phospholipase A2 group VI
PlGF	placental growth factor
PUFAs	polyunsaturated fatty acids
PUFA-PL-OOH	polyunsaturated fatty acid-containing phospholipid hydroperoxide
RAS	renin-angiotensin system
RBC count	red blood cell count
RBCs	red blood cells
RDA	recommended dietary allowance
ROS	reactive oxygen species
SDF-1α	stromal cell–derived factor 1, also known as chemokine CXCL12
sFlt-1	soluble fms-like tyrosine kinase-1, also known as soluble vascular endothelial growth factor receptor 1 (VEGFR-1)
sHJV	soluble hemojuvelin
SLC11A2	gene encoding divalent metal transporter 1 (DMT1)
SLC39A	solute carrier family 39A (known as the ZIP or ZRT- and IRT-like protein family)
SLC39A8	solute carrier family 39A zinc transporter 8, also known as ZIP 8
SLC39A14	solute carrier family 39A zinc transporter 14, also known as ZIP 14
SLC40A1	ferroportin, also known as solute carrier family 40 member 1
SMAD	small mother against decapentaplegic (family of proteins)
SOD2	superoxide dismutase 2
SQSTM1	sequestosome 1, protein p62
STB	syncytiotrophoblast
STEAP	six-transmembrane epithelial antigen of prostate
STEAP3, STEAP4	six-transmembrane epithelial antigen of prostate (STEAP) family members 3 and 4
sVEGFR-1	soluble vascular endothelial growth factor receptor 1, also known as soluble fms-like tyrosine kinase-1 (sFlt-1)
TET	ten-eleven translocation
Tf	transferrin
Tf-Fe (III)2	diferric transferrin
TfR1	transferrin receptor 1
TFRC	gene encoding transferrin receptor protein 1 (TfR1), also known as CD71
TIBC	total iron-binding capacity
TNF-α	tumor necrosis factor alpha
TXNIP	thioredoxin-interacting protein
UTRs	untranslated regions of mRNA transcripts
VEGF	vascular endothelial growth factor
VEGF-A	vascular endothelial growth factor A
VSMC	vascular smooth muscle cell
XO	xanthine oxidase
ZIP14	ZRT/IRT-like protein 14

## References

[1] Brooks S., Frost A., Sanders D.J., Vatne E., Calamoneri T., Stroh E., Jordan A., Nickerson B.S., Saenz C. (2026). Micronutrient intake and predictors of adequacy in female collegiate dancers consuming low-energy diets. J. Int. Soc. Sports Nutr..

[2] Maqbool A., Parks E.P., Shaikhkhalil A., Panganiban J., Mitchell J.A., Stallings V.A., Kliegman R.M., St Geme J.W., Blum N.J., Shah S.S., Tasker R.C., Wilson K.M. (2020). Nutritional requirements. Nelson Textbook of Pediatrics.

[3] Obeagu E.I. (2025). Iron homeostasis and health: Understanding its role beyond blood health—A narrative review. Ann. Med. Surg..

[4] Yiannikourides A., Latunde-Dada G.O. (2019). A Short Review of Iron Metabolism and Pathophysiology of Iron Disorders. Medicines.

[5] Lewkowitz A.K., Tuuli M.G. (2023). Identifying and treating iron deficiency anemia in pregnancy. Hematol. Am. Soc. Hematol. Educ. Program.

[6] Moyle K.A. (2025). A practical review of iron deficiency in pregnancy. Semin. Fetal Neonatal Med..

[7] Mégier C., Peoc’h K., Puy V., Cordier A.G. (2022). Iron Metabolism in Normal and Pathological Pregnancies and Fetal Consequences. Metabolites.

[8] Benson A.E., Shatzel J.J., Ryan K.S., Hedges M.A., Martens K., Aslan J.E., Lo J.O. (2022). The incidence, complications, and treatment of iron deficiency in pregnancy. Eur. J. Haematol..

[9] Azami M., Badfar G., Khalighi Z., Qasemi P., Shohani M., Soleymani A., Abbasalizadeh S. (2019). The association between anemia and postpartum depression: A systematic review and meta-analysis. Casp. J. Intern. Med..

[10] Quezada-Pinedo H.G., Cassel F., Duijts L., Muckenthaler M.U., Gassmann M., Jaddoe V.W.V., Reiss I.K.M., Vermeulen M.J. (2021). Maternal Iron Status in Pregnancy and Child Health Outcomes after Birth: A Systematic Review and Meta-Analysis. Nutrients.

[11] Habe S., Haruna M., Yonezawa K., Usui Y., Sasaki S., Nagamatsu T., Fujita M., Suetsugu Y., Ohori R., Tanaka M. (2024). Factors Associated with Anemia and Iron Deficiency during Pregnancy: A Prospective Observational Study in Japan. Nutrients.

[12] Georgieff M.K., Krebs N.F., Cusick S.E. (2019). The Benefits and Risks of Iron Supplementation in Pregnancy and Childhood. Annu. Rev. Nutr..

[13] Ege S., Akduman H., Aşir A., Korak T., Aşir F. (2025). Altered placental iron transport and putative ferroptosis pathways in pregnancies with excessive gestational weight gain: A prospective cohort study. Medicine.

[14] Liu M., Wu K., Wu Y. (2023). The emerging role of ferroptosis in female reproductive disorders. Biomed. Pharmacother..

[15] Ng S.W., Norwitz S.G., Norwitz E.R. (2019). The Impact of Iron Overload and Ferroptosis on Reproductive Disorders in Humans: Implications for Preeclampsia. Int. J. Mol. Sci..

[16] Yang Y., Zuo H., Ma X., Chen W., Sun M., Xiang Q., Wei Y., Zhao Y., Qi H., Liu T. (2026). Iron Overload-Induced Ferroptosis Drives Placental Dysfunction in Preeclampsia. Hypertension.

[17] Zhong J., Jiang R., Liu N., Cai Q., Cao Q., Du Y., Zhao H. (2025). Iron-Immune Crosstalk at the Maternal-Fetal Interface: Emerging Mechanisms in the Pathogenesis of Preeclampsia. Antioxidants.

[18] Thalapalliyil K., Bobby Z., Dorairajan G., Toi P.C. (2026). Placental iron-driven oxidative imbalance and changes in iron homeostasis in diabetes-in-pregnancy: A cross-sectional analytical study. Placenta.

[19] Shen X., Obore N., Wang Y., Yu T., Yu H. (2023). The Role of Ferroptosis in Placental-Related Diseases. Reprod. Sci..

[20] Cao C., Fleming M.D. (2016). The placenta: The forgotten essential organ of iron transport. Nutr. Rev..

[21] Sangkhae V., Fisher A.L., Ganz T., Nemeth E. (2023). Iron Homeostasis During Pregnancy: Maternal, Placental, and Fetal Regulatory Mechanisms. Annu. Rev. Nutr..

[22] Sangkhae V., Nemeth E. (2019). Placental iron transport: The mechanism and regulatory circuits. Free Radic. Biol. Med..

[23] O’Brien K.O. (2022). Maternal, fetal and placental regulation of placental iron trafficking. Placenta.

[24] Ru Q., Li Y., Chen L., Wu Y., Min J., Wang F. (2024). Iron homeostasis and ferroptosis in human diseases: Mechanisms and therapeutic prospects. Signal Transduct. Target. Ther..

[25] Tandara L., Salamunic I. (2012). Iron metabolism: Current facts and future directions. Biochem. Med..

[26] Oliveira F., Rocha S., Fernandes R. (2014). Iron metabolism: From health to disease. J. Clin. Lab. Anal..

[27] Roy R., Kück M., Radziwolek L., Kerling A. (2022). Iron Deficiency in Adolescent and Young Adult German Athletes-A Retrospective Study. Nutrients.

[28] Silva B., Faustino P. (2015). An overview of molecular basis of iron metabolism regulation and the associated pathologies. Biochim. Biophys. Acta.

[29] Kolarš B., Mijatović Jovin V., Živanović N., Minaković I., Gvozdenović N., Dickov Kokeza I., Lesjak M. (2025). Iron Deficiency and Iron Deficiency Anemia: A Comprehensive Overview of Established and Emerging Concepts. Pharmaceuticals.

[30] Beard J.L. (2001). Iron biology in immune function, muscle metabolism and neuronal functioning. J. Nutr..

[31] Lal A. (2020). Iron in Health and Disease: An Update. Indian. J. Pediatr..

[32] Coffey R., Ganz T. (2017). Iron homeostasis: An anthropocentric perspective. J. Biol. Chem..

[33] Ganz T. (2013). Systemic iron homeostasis. Physiol. Rev..

[34] Dlouhy A.C., Outten C.E. (2013). The iron metallome in eukaryotic organisms. Met. Ions Life Sci..

[35] Philpott C.C., Protchenko O., Wang Y., Novoa-Aponte L., Leon-Torres A., Grounds S., Tietgens A.J. (2023). Iron-tracking strategies: Chaperones capture iron in the cytosolic labile iron pool. Front. Mol. Biosci..

[36] Lazarte S.S., Mónaco M.E., Jimenez C.L., Ledesma Achem M.E., Terán M.M., Issé B.A. (2015). Erythrocyte Catalase Activity in More Frequent Microcytic Hypochromic Anemia: Beta-Thalassemia Trait and Iron Deficiency Anemia. Adv. Hematol..

[37] Alfonso-Prieto M., Vidossich P., Rovira C. (2012). The reaction mechanisms of heme catalases: An atomistic view by ab initio molecular dynamics. Arch. Biochem. Biophys..

[38] Saito H. (2019). Storage Iron Turnover from a New Perspective. Acta Haematol..

[39] Saito H., Tomita A., Ohashi H., Maeda H., Hayashi H., Naoe T. (2012). Determination of ferritin and hemosiderin iron in patients with normal iron stores and iron overload by serum ferritin kinetics. Nagoya J. Med. Sci..

[40] Porter J., Cappellini M.D., Farmakis D., Porter J., Taher A., Kattamis A. (2023). Iron overload: Pathophysiology, diagnosis and monitoring. 2021 Guidelines: For the Management of Transfusion Dependent Thalassaemia (TDT) [Internet].

[41] Pinyopornpanish K., Tantiworawit A., Leerapun A., Soontornpun A., Thongsawat S. (2023). Secondary Iron Overload and the Liver: A Comprehensive Review. J. Clin. Transl. Hepatol..

[42] Roemhild K., von Maltzahn F., Weiskirchen R., Knüchel R., von Stillfried S., Lammers T. (2021). Iron metabolism: Pathophysiology and pharmacology. Trends Pharmacol. Sci..

[43] Wang C.Y., Babitt J.L. (2019). Liver iron sensing and body iron homeostasis. Blood.

[44] Ogun A.S., Adeyinka A. (2026). Biochemistry, Transferrin. [Updated 7 July 2025]. StatPearls [Internet].

[45] Baddam S., Chen R.J. (2026). Iron Overload and Toxicity. [Updated 7 July 2025]. StatPearls [Internet].

[46] Kontoghiorghes G.J. (2023). Iron Load Toxicity in Medicine: From Molecular and Cellular Aspects to Clinical Implications. Int. J. Mol. Sci..

[47] Weichhart T. (2024). Transferrin: The iron transporter takes control. Blood.

[48] Lv H., Shang P. (2018). The significance, trafficking and determination of labile iron in cytosol, mitochondria and lysosomes. Metallomics.

[49] Philpott C.C., Jadhav S. (2019). The ins and outs of iron: Escorting iron through the mammalian cytosol. Free Radic. Biol. Med..

[50] Huang Q., Ma C., Wei P., Wang Q., Ren H., Li X. (2026). Advances in Fenton reaction-mediated ferroptosis for enhanced cancer therapy: Mechanisms, amplification strategies, and synergistic approaches. J. Mater. Chem. B.

[51] Camaschella C., Nai A., Silvestri L. (2020). Iron metabolism and iron disorders revisited in the hepcidin era. Haematologica.

[52] Qiu L., Frazer D.M., Hu M., Song R., Liu X., Qin X., Ma J., Zhou J., Tan Z., Ren F. (2025). Mechanism and regulation of iron absorption throughout the life cycle. J. Adv. Res..

[53] Galy B., Ferring-Appel D., Becker C., Gretz N., Gröne H.J., Schümann K., Hentze M.W. (2013). Iron regulatory proteins control a mucosal block to intestinal iron absorption. Cell Rep..

[54] Qatato M., Bonadonna M., Palais G., Ertl A., Schmidt G., Polycarpou-Schwarz M., Karim Z., Galy B. (2022). IRE-dependent Regulation of Intestinal Dmt1 Prevails During Chronic Dietary Iron Deficiency but is Dispensable in Conditions of Acute Erythropoietic Stress. Hemasphere.

[55] Wilkinson N., Pantopoulos K. (2014). The IRP/IRE system in vivo: Insights from mouse models. Front. Pharmacol..

[56] Tsuji Y., Ninomiya-Tsuji J., Shen M.Y.F., DiFrancesco B.R. (2025). Modulation of iron metabolism by new chemicals interacting with the iron regulatory system. Redox Biol..

[57] Bouton C., Oliveira L., Drapier J.C. (1998). Converse modulation of IRP1 and IRP2 by immunological stimuli in murine RAW 264.7 macrophages. J. Biol. Chem..

[58] Khan M.A., Mohammad T., Malik A., Hassan M.I., Domashevskiy A.V. (2023). Iron response elements (IREs)-mRNA of Alzheimer’s amyloid precursor protein binding to iron regulatory protein (IRP1): A combined molecular docking and spectroscopic approach. Sci. Rep..

[59] Zhang D.L., Ghosh M.C., Rouault T.A. (2014). The physiological functions of iron regulatory proteins in iron homeostasis—An update. Front. Pharmacol..

[60] Garza K.R., Clarke S.L., Ho Y.H., Bruss M.D., Vasanthakumar A., Anderson S.A., Eisenstein R.S. (2020). Differential translational control of 5′ IRE-containing mRNA in response to dietary iron deficiency and acute iron overload. Metallomics.

[61] Connell G.J., Abasiri I.M., Chaney E.H. (2023). A temporal difference in the stabilization of two mRNAs with a 3′ iron-responsive element during iron deficiency. RNA.

[62] Nemeth E., Ganz T. (2021). Hepcidin-Ferroportin Interaction Controls Systemic Iron Homeostasis. Int. J. Mol. Sci..

[63] Parreira L.F., Teixeira A.V.R., Siqueira S.S.N., Siqueira N.S.N., de Castro M.M., Genaro L.M., Pereira I.M., Gallina N.C., Leal R.F. (2026). The role of hepcidin in the inflammatory and iron homeostasis axis in inflammatory bowel diseases: A systematic review. Transl. Gastroenterol. Hepatol..

[64] Zheng Q., Li Y., Li J. (2026). Current research on the HIF-2α-EPO-Hb axis in hypoxic environments: From molecular mechanisms to clinical. Front. Med..

[65] Vogt A.S., Arsiwala T., Mohsen M., Vogel M., Manolova V., Bachmann M.F. (2021). On Iron Metabolism and Its Regulation. Int. J. Mol. Sci..

[66] Yanatori I., Kishi F. (2019). DMT1 and iron transport. Free Radic. Biol. Med..

[67] Taylor M., Qu A., Anderson E.R., Matsubara T., Martin A., Gonzalez F.J., Shah Y.M. (2011). Hypoxia-inducible factor-2α mediates the adaptive increase of intestinal ferroportin during iron deficiency in mice. Gastroenterology.

[68] Arezes J., Foy N., McHugh K., Sawant A., Quinkert D., Terraube V., Brinth A., Tam M., LaVallie E.R., Taylor S. (2018). Erythroferrone inhibits the induction of hepcidin by BMP6. Blood.

[69] Silvestri L., Nai A., Dulja A., Pagani A. (2019). Hepcidin and the BMP-SMAD pathway: An unexpected liaison. Vitam. Horm..

[70] Xiao X., Alfaro-Magallanes V.M., Babitt J.L. (2020). Bone morphogenic proteins in iron homeostasis. Bone.

[71] Schwartz A.J., Das N.K., Ramakrishnan S.K., Jain C., Jurkovic M.T., Wu J., Nemeth E., Lakhal-Littleton S., Colacino J.A., Shah Y.M. (2019). Hepatic hepcidin/intestinal HIF-2α axis maintains iron absorption during iron deficiency and overload. J. Clin. Investig..

[72] Wilkinson N., Pantopoulos K. (2013). IRP1 regulates erythropoiesis and systemic iron homeostasis by controlling HIF2α mRNA translation. Blood.

[73] Anderson S.A., Nizzi C.P., Chang Y.I., Deck K.M., Schmidt P.J., Galy B., Damnernsawad A., Broman A.T., Kendziorski C., Hentze M.W. (2013). The IRP1-HIF-2α axis coordinates iron and oxygen sensing with erythropoiesis and iron absorption. Cell Metab..

[74] Ghosh M.C., Zhang D.L., Jeong S.Y., Kovtunovych G., Ollivierre-Wilson H., Noguchi A., Tu T., Senecal T., Robinson G., Crooks D.R. (2013). Deletion of iron regulatory protein 1 causes polycythemia and pulmonary hypertension in mice through translational derepression of HIF2α. Cell Metab..

[75] Skolmowska D., Głąbska D. (2019). Analysis of Heme and Non-Heme Iron Intake and Iron Dietary Sources in Adolescent Menstruating Females in a National Polish Sample. Nutrients.

[76] De Martin S. (2026). Beyond bioavailability: Dual-source iron improves absorption and cellular handling. Front. Nutr..

[77] West A.R., Oates P.S. (2008). Mechanisms of heme iron absorption: Current questions and controversies. World J. Gastroenterol..

[78] Sharp P., Srai S.K. (2007). Molecular mechanisms involved in intestinal iron absorption. World J. Gastroenterol..

[79] Yanatori I., Tabuchi M., Kawai Y., Yasui Y., Akagi R., Kishi F. (2010). Heme and non-heme iron transporters in non-polarized and polarized cells. BMC Cell Biol..

[80] Pope S.R., McBride M.J., Nair M.A., Salas-Solá X.E., Krebs C., Bollinger J.M., Boal A.K. (2025). Heme Oxygenase-Like Metalloenzymes. Annu. Rev. Biochem..

[81] Latunde-Dada G.O., Simpson R.J., McKie A.T. (2006). Recent advances in mammalian haem transport. Trends Biochem. Sci..

[82] Vanoaica L., Darshan D., Richman L., Schümann K., Kühn L.C. (2010). Intestinal ferritin H is required for an accurate control of iron absorption. Cell Metab..

[83] Andrews N.C. (2010). Ferrit(in)ing out new mechanisms in iron homeostasis. Cell Metab..

[84] Guo Q., Qian C., Wang X., Qian Z.M. (2025). Transferrin receptors. Exp. Mol. Med..

[85] Yu Y., Jiang L., Wang H., Shen Z., Cheng Q., Zhang P., Wang J., Wu Q., Fang X., Duan L. (2020). Hepatic transferrin plays a role in systemic iron homeostasis and liver ferroptosis. Blood.

[86] Rouault T.A. (2002). Post-transcriptional regulation of human iron metabolism by iron regulatory proteins. Blood Cells Mol. Dis..

[87] Charlebois E., Fillebeen C., Katsarou A., Rabinovich A., Wisniewski K., Venkataramani V., Michalke B., Velentza A., Pantopoulos K. (2022). A crosstalk between hepcidin and IRE/IRP pathways controls ferroportin expression and determines serum iron levels in mice. eLife.

[88] Miyazawa M., Bogdan A.R., Hashimoto K., Tsuji Y. (2018). Regulation of transferrin receptor-1 mRNA by the interplay between IRE-binding proteins and miR-7/miR-141 in the 3′-IRE stem-loops. RNA.

[89] Saito H. (2024). Nature of storage iron turnover. Nagoya J. Med. Sci..

[90] Enko D. (2025). Physiology of Iron Metabolism. Clin. Lab..

[91] Li W., Fredriksson M., Dernroth D.N., Mahmod H., Mai X.M., Östgren C.J., Yuan X.M. (2026). Sex differences in iron stores and associations of body iron with cardiovascular risk factors in the middle-aged general population. Front. Endocrinol..

[92] Beutler E. (2007). Iron storage disease: Facts, fiction and progress. Blood Cells Mol. Dis..

[93] Hsu C.C., Senussi N.H., Fertrin K.Y., Kowdley K.V. (2022). Iron overload disorders. Hepatol. Commun..

[94] Ahmed S., Peterson S.J., Parikh M.A., Frishman W.H. (2025). Cardiovascular Manifestations of Hemochromatosis: A Review of Pathophysiology, Mechanisms, and Treatment Options. Cardiol. Rev..

[95] Griffiths W.J.H., Besser M., Bowden D.J., Kelly D.A. (2021). Juvenile haemochromatosis. Lancet Child. Adolesc. Health.

[96] Badulescu O.-V., Cosmin Tudor R., Filip N., Bordeianu G., Ciocoiu M., Bararu-Bojan I., Vladeanu M.C., Frasinariu O.E., Barbosu L.I., Tesoi D.F. (2025). Iron Metabolism and Hemochromatosis: Pathogenic Pathways to Multiple Organ Dysfunction—Clinical and Therapeutic Implications. Human Iron Metabolism—Biology, Health, and Disease.

[97] Batts K.P. (2007). Iron overload syndromes and the liver. Mod. Pathol..

[98] Kasztura M., Kiczak L., Pasławska U., Bania J., Janiszewski A., Tomaszek A., Zacharski M., Noszczyk-Nowak A., Pasławski R., Tabiś A. (2022). Hemosiderin Accumulation in Liver Decreases Iron Availability in Tachycardia-Induced Porcine Congestive Heart Failure Model. Int. J. Mol. Sci..

[99] Soe-Lin S., Apte S.S., Andriopoulos B., Andrews M.C., Schranzhofer M., Kahawita T., Garcia-Santos D., Ponka P. (2009). Nramp1 promotes efficient macrophage recycling of iron following erythrophagocytosis in vivo. Proc. Natl. Acad. Sci. USA.

[100] Jormakka M. (2023). Structural insights into ferroportin mediated iron transport. Biochem. Soc. Trans..

[101] White C., Yuan X., Schmidt P.J., Bresciani E., Samuel T.K., Campagna D., Hall C., Bishop K., Calicchio M.L., Lapierre A. (2013). HRG1 is essential for heme transport from the phagolysosome of macrophages during erythrophagocytosis. Cell Metab..

[102] Ryter S.W. (2020). Therapeutic Potential of Heme Oxygenase-1 and Carbon Monoxide in Acute Organ Injury, Critical Illness, and Inflammatory Disorders. Antioxidants.

[103] Korolnek T., Hamza I. (2015). Macrophages and iron trafficking at the birth and death of red cells. Blood.

[104] Kovtunovych G., Eckhaus M.A., Ghosh M.C., Ollivierre-Wilson H., Rouault T.A. (2010). Dysfunction of the heme recycling system in heme oxygenase 1-deficient mice: Effects on macrophage viability and tissue iron distribution. Blood.

[105] Starzyński R.R., Canonne-Hergaux F., Lenartowicz M., Krzeptowski W., Willemetz A., Styś A., Bierła J., Pietrzak P., Dziaman T., Lipiński P. (2013). Ferroportin expression in haem oxygenase 1-deficient mice. Biochem. J..

[106] Zurawska G., Sas Z., Jończy A., Mahadeva R., Slusarczyk P., Chwałek M., Seehofer D., Damm G., Mazgaj R., Skórzyński M. (2026). Liver sinusoidal endothelial cells constitute a major route for hemoglobin clearance. EMBO Rep..

[107] Charlebois E., Fillebeen C., Presley J., Cagnone G., Lisi V., Lavallée V.P., Joyal J.S., Pantopoulos K. (2023). Liver sinusoidal endothelial cells induce BMP6 expression in response to non-transferrin-bound iron. Blood.

[108] Qu J., Wang L., Li Y., Li X. (2024). Liver sinusoidal endothelial cell: An important yet often overlooked player in the liver fibrosis. Clin. Mol. Hepatol..

[109] Shetty S., Lalor P.F., Adams D.H. (2018). Liver sinusoidal endothelial cells—Gatekeepers of hepatic immunity. Nat. Rev. Gastroenterol. Hepatol..

[110] Poisson J., Lemoinne S., Boulanger C., Durand F., Moreau R., Valla D., Rautou P.E. (2017). Liver sinusoidal endothelial cells: Physiology and role in liver diseases. J. Hepatol..

[111] Parrow N.L., Fleming R.E. (2017). Liver sinusoidal endothelial cells as iron sensors. Blood.

[112] Hamad M., Bajbouj K., Taneera J. (2020). The Case for an Estrogen-iron Axis in Health and Disease. Exp. Clin. Endocrinol. Diabetes.

[113] Satué K., Fazio E., Medica P. (2023). Estrogen-iron axis in cyclic mares: Effect of age. Theriogenology.

[114] Zhang V., Fisher A.L., Hewitt M.S., Ganz T., Nemeth E., Sangkhae V. (2024). Maternal prolactin or estrogen signaling in hepatocytes does not regulate iron homeostasis during pregnancy. Haematologica.

[115] Ikeda Y., Tajima S., Izawa-Ishizawa Y., Kihira Y., Ishizawa K., Tomita S., Tsuchiya K., Tamaki T. (2012). Estrogen regulates hepcidin expression via GPR30-BMP6-dependent signaling in hepatocytes. PLoS ONE.

[116] Chen S., Xiao J., Zhou S., Wumiti T., Zhao Z., Zhao R., Pan Y., Wang Q., Ma Y., Wu L. (2025). The GPR30-Mediated BMP-6/HEP/FPN Signaling Pathway Inhibits Ferroptosis in Bone Marrow Mesenchymal Stem Cells to Alleviate Osteoporosis. Int. J. Mol. Sci..

[117] Kopeć Z., Starzyński R.R., Jończy A., Mazgaj R., Lipiński P. (2021). Role of Iron Metabolism-Related Genes in Prenatal Development: Insights from Mouse Transgenic Models. Genes.

[118] Zhang P., Wang S., Wang L., Shan B.C., Zhang H., Yang F., Zhou Z.Q., Wang X., Yuan Y., Xu Y.J. (2018). Hepcidin is an endogenous protective factor for osteoporosis by reducing iron levels. J. Mol. Endocrinol..

[119] Chen B., Li G.F., Shen Y., Huang X.I., Xu Y.J. (2015). Reducing iron accumulation: A potential approach for the prevention and treatment of postmenopausal osteoporosis. Exp. Ther. Med..

[120] Jian J., Pelle E., Huang X. (2009). Iron and menopause: Does increased iron affect the health of postmenopausal women?. Antioxid. Redox Signal.

[121] Yang Y., Zhang X., Yang Y., Gao P., Fan W., Zheng T., Yang W., Tang Y., Cai K. (2024). A two-pronged approach to inhibit ferroptosis of MSCs caused by the iron overload in postmenopausal osteoporosis and promote osseointegration of titanium implant. Bioact. Mater..

[122] Fisher A.L., Nemeth E. (2017). Iron homeostasis during pregnancy. Am. J. Clin. Nutr..

[123] Koenig M.D., Tussing-Humphreys L., Day J., Cadwell B., Nemeth E. (2014). Hepcidin and iron homeostasis during pregnancy. Nutrients.

[124] Millard K.N., Frazer D.M., Wilkins S.J., Anderson G.J. (2004). Changes in the expression of intestinal iron transport and hepatic regulatory molecules explain the enhanced iron absorption associated with pregnancy in the rat. Gut.

[125] Nemeth E., Ganz T. (2023). Hepcidin and Iron in Health and Disease. Annu. Rev. Med..

[126] Helman S.L., Wilkins S.J., Chan J.C.J., Hartel G., Wallace D.F., Anderson G.J., Frazer D.M. (2023). A Decrease in Maternal Iron Levels Is the Predominant Factor Suppressing Hepcidin during Pregnancy in Mice. Int. J. Mol. Sci..

[127] Bedran M., Manceau H., Ollivier H., Deleschaux C., Beaufrère A., Ben M’Barek I., Peoc’h K. (2026). Pregnancy and iron: Bridging metabolic demands and supplementation needs. Clin. Nutr. ESPEN.

[128] Dogu S.Y., Acar H., Atalmis H.A., Guler E.Y., Bafali I.O., Guzelbag B., Ocal A., Guleroglu F.Y., Yilmaz I., Cetin A. (2026). Iron supplementation-induced modulation of hepcidin, erythroferrone, and oxidative balance in iron-deficient pregnant women. BMC Pregnancy Childbirth.

[129] Fielding K.L., Bennett C., Pettikiriarachchi A., Jones N., Harding R., McLean A.R.D., Randall L., Schaeper U., Martinez A., Ataíde R. (2025). Maternal Tmprss6 is required for hepcidin suppression and fetal health. Blood.

[130] Fauzan R., Defrin Susmiati Lipoeto N.I. (2025). Impact of TMPRSS6 Genetic Variants on Maternal Iron Status in Pregnancy: A Systematic Review. Birth Defects Res..

[131] Lee P. (2009). Role of matriptase-2 (TMPRSS6) in iron metabolism. Acta Haematol..

[132] Satué K., Fazio E., La Fauci D., Bruschetta G., Medica P. (2023). Adaptive Response of Estrogen-Iron Axis in Pregnant Purebred Spanish Mares of Different Age. J. Equine Vet. Sci..

[133] Kratz A., Ferraro M., Sluss P.M., Lewandrowski K.B. (2004). Case records of the Massachusetts General Hospital. Weekly clinicopathological exercises. Laboratory reference values. N. Engl. J. Med..

[134] Kasvosve I., Delanghe J. (2002). Total iron binding capacity and transferrin concentration in the assessment of iron status. Clin. Chem. Lab. Med..

[135] Abbassi-Ghanavati M., Greer L.G., Cunningham F.G. (2009). Pregnancy and laboratory studies: A reference table for clinicians. Obstet. Gynecol..

[136] Ritchie R.F., Palomaki G.E., Neveux L.M., Navolotskaia O., Ledue T.B., Craig W.Y. (2002). Reference distributions for serum iron and transferrin saturation: A comparison of a large cohort to the world’s literature. J. Clin. Lab. Anal..

[137] Paradkar M.N., Mejia I., Abraheem R., Marroquín León E., Firdous A., Barroso M.J., Sampathkumar D.K., Morani Z. (2024). Assessing the Impact of Hematological Changes in Pregnancy on Maternal and Fetal Death: A Narrative Review. Cureus.

[138] González-Chávez S.A., Arévalo-Gallegos S., Rascón-Cruz Q. (2009). Lactoferrin: Structure, function and applications. Int. J. Antimicrob. Agents..

[139] Hao L., Shan Q., Wei J., Ma F., Sun P. (2019). Lactoferrin: Major Physiological Functions and Applications. Curr. Protein Pept. Sci..

[140] Cheng R., Tang X., Huang X., Dong X., Chen Y., Gao J., Bai L., Tadese D.A., Zhao Q., Li M. (2026). Estrogen upregulates lactoferrin to induce hypercoagulability for hemostatic protection during pregnancy. Blood.

[141] Soares M.J., Varberg K.M., Iqbal K. (2018). Hemochorial placentation: Development, function, and adaptations. Biol. Reprod..

[142] Zych-Krekora K., Sylwestrzak O., Krekora M. (2025). The Critical Role of Iron in Pregnancy, Puerperium, and Fetal Development. J. Clin. Med..

[143] Lakhal-Littleton S. (2021). Advances in understanding the crosstalk between mother and fetus on iron utilization. Semin. Hematol..

[144] Balesaria S., Hanif R., Salama M.F., Raja K., Bayele H.K., McArdle H., Srai S.K. (2012). Fetal iron levels are regulated by maternal and fetal Hfe genotype and dietary iron. Haematologica.

[145] Santhakumar S., Edison E.S. (2024). Molecular insights into placental iron transfer mechanisms and maternofetal regulation. Arch. Gynecol. Obstet..

[146] Zaugg J., Solenthaler F., Albrecht C. (2022). Materno-fetal iron transfer and the emerging role of ferroptosis pathways. Biochem. Pharmacol..

[147] Delaney K.M., Guillet R., Pressman E.K., Caulfield L.E., Zavaleta N., Abrams S.A., O’Brien K.O. (2020). Iron absorption during pregnancy is underestimated when iron utilization by the placenta and fetus is ignored. Am. J. Clin. Nutr..

[148] Hafez S. (2017). Comparative Placental Anatomy: Divergent Structures Serving a Common Purpose. Prog. Mol. Biol. Transl. Sci..

[149] Cindrova-Davies T., Sferruzzi-Perri A.N. (2022). Human placental development and function. Semin. Cell Dev. Biol..

[150] Kojima J., Ono M., Kuji N., Nishi H. (2022). Human Chorionic Villous Differentiation and Placental Development. Int. J. Mol. Sci..

[151] Krüger-Genge A., Blocki A., Franke R.P., Jung F. (2019). Vascular Endothelial Cell Biology: An Update. Int. J. Mol. Sci..

[152] Kalisch-Smith J.I. (2025). Feto-placental blood vessel development. Development.

[153] Palaiologou E., Etter O., Goggin P., Chatelet D.S., Johnston D.A., Lofthouse E.M., Doherty R., Pearson-Farr J., Sengers B.G., Torrens C. (2020). Human placental villi contain stromal macrovesicles associated with networks of stellate cells. J. Anat..

[154] Arora M., Mehndiratta M., Almeida E.A., Kotru M., Gupta B. (2024). Gene expression of iron transporters, markers of vascularization and structural integrity in placenta of mothers with and without anemia. Mol. Biol. Rep..

[155] Bu J.T., Bartnikas T.B. (2015). The use of hypotransferrinemic mice in studies of iron biology. Biometals.

[156] Gammella E., Lomoriello I.S., Conte A., Freddi S., Alberghini A., Poli M., Sigismund S., Cairo G., Recalcati S. (2021). Unconventional endocytosis and trafficking of transferrin receptor induced by iron. Mol. Biol. Cell..

[157] Zhang Y., Lu Y., Jin L. (2022). Iron Metabolism and Ferroptosis in Physiological and Pathological Pregnancy. Int. J. Mol. Sci..

[158] Dautry-Varsat A., Ciechanover A., Lodish H.F. (1983). pH and the recycling of transferrin during receptor-mediated endocytosis. Proc. Natl. Acad. Sci. USA.

[159] Santhakumar S., Athiyarath R., Cherian A.G., Abraham V.J., George B., Lipiński P., Edison E.S. (2023). Impact of maternal iron deficiency anemia on fetal iron status and placental iron transporters in human pregnancy. Blood Cells Mol. Dis..

[160] Li Y.Q., Bai B., Cao X.X., Zhang Y.H., Yan H., Zheng Q.Q., Zhuang G.H. (2012). Divalent metal transporter 1 expression and regulation in human placenta. Biol. Trace Elem. Res..

[161] Helman S.L., Zhou J., Fuqua B.K., Lu Y., Collins J.F., Chen H., Vulpe C.D., Anderson G.J., Frazer D.M. (2023). The biology of mammalian multi-copper ferroxidases. Biometals.

[162] Helman S.L., Wilkins S.J., McKeating D.R., Perkins A.V., Whibley P.E., Cuffe J.S.M., Simmons D.G., Fuqua B.K., Vulpe C.D., Wallace D.F. (2021). The Placental Ferroxidase Zyklopen Is Not Essential for Iron Transport to the Fetus in Mice. J. Nutr..

[163] Ji S., Gumina D., McPeak K., Moldovan R., Post M.D., Su E.J. (2021). Human placental villous stromal extracellular matrix regulates fetoplacental angiogenesis in severe fetal growth restriction. Clin. Sci..

[164] Ramadoss P., Chiappini F., Bilban M., Hollenberg A.N. (2010). Regulation of hepatic six transmembrane epithelial antigen of prostate 4 (STEAP4) expression by STAT3 and CCAAT/enhancer-binding protein alpha. J. Biol. Chem..

[165] McCarthy R.C., Kosman D.J. (2015). Mechanisms and regulation of iron trafficking across the capillary endothelial cells of the blood-brain barrier. Front. Mol. Neurosci..

[166] Hvidberg V., Maniecki M.B., Jacobsen C., Højrup P., Møller H.J., Moestrup S.K. (2005). Identification of the receptor scavenging hemopexin-heme complexes. Blood.

[167] Cao C., Pressman E.K., Cooper E.M., Guillet R., Westerman M., O’Brien K.O. (2014). Placental heme receptor LRP1 correlates with the heme exporter FLVCR1 and neonatal iron status. Reproduction.

[168] Jaacks L.M., Young M.F., Essley B.V., McNanley T.J., Cooper E.M., Pressman E.K., McIntyre A.W., Orlando M.S., Abkowitz J.L., Guillet R. (2011). Placental expression of the heme transporter, feline leukemia virus subgroup C receptor, is related to maternal iron status in pregnant adolescents. J. Nutr..

[169] Le Blanc S., Garrick M.D., Arredondo M. (2012). Heme carrier protein 1 transports heme and is involved in heme-Fe metabolism. Am. J. Physiol. Cell Physiol..

[170] Chong W.S., Kwan P.C., Chan L.Y., Chiu P.Y., Cheung T.K., Lau T.K. (2005). Expression of divalent metal transporter 1 (DMT1) isoforms in first trimester human placenta and embryonic tissues. Hum. Reprod..

[171] Inoue R., Irie Y., Akagi R. (2021). Role of heme oxygenase-1 in human placenta on iron supply to fetus. Placenta.

[172] Bainbridge S.A., Smith G.N. (2005). HO in pregnancy. Free Radic. Biol. Med..

[173] Sangkhae V., Fisher A.L., Wong S., Koenig M.D., Tussing-Humphreys L., Chu A., Lelić M., Ganz T., Nemeth E. (2020). Effects of maternal iron status on placental and fetal iron homeostasis. J. Clin. Investig..

[174] Girijashanker K., He L., Soleimani M., Reed J.M., Li H., Liu Z., Wang B., Dalton T.P., Nebert D.W. (2008). Slc39a14 gene encodes ZIP14, a metal/bicarbonate symporter: Similarities to the ZIP8 transporter. Mol. Pharmacol..

[175] Schroeder M., Yi N., Fuenzalida B., Furrer T.C., du Toit T., Mueller M., Ontsouka E., Albrecht C. (2026). Placental iron transport under maternal stress: A missing link in foetal programming and mental health. EBioMedicine.

[176] McDonald E.A., Gundogan F., Olveda R.M., Bartnikas T.B., Kurtis J.D., Friedman J.F. (2022). Iron transport across the human placenta is regulated by hepcidin. Pediatr. Res..

[177] Lai S., Yu W., Liu Y., Yang Y., Zhang X. (2024). Current research and evidence gaps on placental development in iron deficiency anemia. Open Life Sci..

[178] Means R.T. (2020). Iron Deficiency and Iron Deficiency Anemia: Implications and Impact in Pregnancy, Fetal Development, and Early Childhood Parameters. Nutrients.

[179] Obeagu G.U., Obeagu E.I. (2025). Complications of anemia in pregnancy: An updated overview for healthcare professionals. Medicine.

[180] Li S., Dempsey N., Gao S., Su S., Xie S., Yao Z., Hu M., Li J., Li Z., Zhang E. (2026). Maternal Haemoglobin and Haematocrit During Pregnancy and Stillbirth Risk: A Prospective, Population-Based Birth Cohort Study. Paediatr. Perinat. Epidemiol..

[181] He F., Liu C. (2026). The influence of maternal anemia on neonatal neurodevelopment: A systematic review and meta-analysis. Asian Biomed. (Res. Rev. News).

[182] Georgieff M.K. (2026). The Effects of Maternal Iron Deficiency and Iron Deficiency Anemia on the Developing Fetal and Neonatal Brain. J. Obstet. Gynaecol. Can..

[183] Soma-Pillay P., Nelson-Piercy C., Tolppanen H., Mebazaa A. (2016). Physiological changes in pregnancy. Cardiovasc. J. Afr..

[184] Fite M.B., Bikila D., Habtu W., Tura A.K., Yadeta T.A., Oljira L., Roba K.T. (2022). Beyond hemoglobin: Uncovering iron deficiency and iron deficiency anemia using serum ferritin concentration among pregnant women in eastern Ethiopia: A community-based study. BMC Nutr..

[185] Babah O.A., Chieme C.F., Abioye A.I., Spaine S.O., Adeyemo T.A., Afolabi B.B. (2025). Red blood cell indices versus serum ferritin as surrogate markers of iron deficiency during pregnancy. PLoS ONE.

[186] Pavord S., Daru J., Prasannan N., Robinson S., Stanworth S., Girling J., BSH Committee (2020). UK guidelines on the management of iron deficiency in pregnancy. Br. J. Haematol..

[187] Peña-Rosas J.P., De-Regil L.M., Gomez Malave H., Flores-Urrutia M.C., Dowswell T. (2015). Intermittent oral iron supplementation during pregnancy. Cochrane Database Syst. Rev..

[188] Milman N.T. (2023). Effects of iron supplementation to iron depleted and iron replete pregnant Danish women: Defining criteria for identification of women who can manage without supplements: A randomized, placebo-controlled study. J. Neonatal Perinat. Med..

[189] Ubom A.E., Begum F., Ramasauskaite D., Nieto-Calvache A.J., Oguttu M., Nunes I., Malel Z.J., Beyeza-Kashesya J., Wright A., FIGO Committee on Childbirth and Postpartum Hemorrhage (2025). FIGO good practice recommendations on anemia in pregnancy, to reduce the incidence and impact of postpartum hemorrhage (PPH). Int. J. Gynaecol. Obstet..

[190] Tsikouras P., Niesigk B., von Tempelhoff G.F., Rath W., Schelkunov O., Daragó P., Csorba R. (2018). Blood rheology during normal pregnancy. Clin. Hemorheol. Microcirc..

[191] Stangret A., Skoda M., Wnuk A., Pyzlak M., Szukiewicz D. (2017). Mild anemia during pregnancy upregulates placental vascularity development. Med. Hypotheses.

[192] Li Q., Wei X., Zhang Y., Zeng W., Lin Y. (2025). Trophoblast adaptation to hypoxia: Balance and dysfunction. Cell Commun. Signal.

[193] Zhao H., Wong R.J., Stevenson D.K. (2021). The Impact of Hypoxia in Early Pregnancy on Placental Cells. Int. J. Mol. Sci..

[194] Stangret A., Wnuk A., Szewczyk G., Pyzlak M., Szukiewicz D. (2017). Maternal hemoglobin concentration and hematocrit values may affect fetus development by influencing placental angiogenesis. J. Matern. Fetal Neonatal Med..

[195] Ahrens S., Singer D. (2025). Placental Adaptation to Hypoxia: The Case of High-Altitude Pregnancies. Int. J. Environ. Res. Public Health.

[196] Patel J., Landers K., Mortimer R.H., Richard K. (2010). Regulation of hypoxia inducible factors (HIF) in hypoxia and normoxia during placental development. Placenta.

[197] Raja F., Chitsaz M., Kundrapu S. Iron Deficiency Anemia. PathologyOutlines.com Website. https://www.pathologyoutlines.com/topic/hematologyirondefanemia.html.

[198] Malinowski A.K., Murji A. (2021). Iron deficiency and iron deficiency anemia in pregnancy. CMAJ.

[199] Silvestro S., Calcaterra V., Pelizzo G., Bramanti P., Mazzon E. (2020). Prenatal Hypoxia and Placental Oxidative Stress: Insights from Animal Models to Clinical Evidences. Antioxidants.

[200] Vornic I., Buciu V., Furau C.G., Gaje P.N., Ceausu R.A., Dumitru C.S., Barb A.C., Novacescu D., Cumpanas A.A., Latcu S.C. (2024). Oxidative Stress and Placental Pathogenesis: A Contemporary Overview of Potential Biomarkers and Emerging Therapeutics. Int. J. Mol. Sci..

[201] Figueiredo Ornelas Braz J.J., Amancio Coelho L.S., Santos Lima D.S., Dias Peixoto M.F., Augusto Bastos Peluso A., de Sá Martins de Bessa A., de Abreu G.R., Souza Santos R.A., Endlich P.W. (2026). Oxidative and antioxidant systems in the placental arterioles of pregnant women with preeclampsia. Eur. J. Obstet. Gynecol. Reprod. Biol..

[202] Grzeszczak K., Łanocha-Arendarczyk N., Malinowski W., Ziętek P., Kosik-Bogacka D. (2023). Oxidative Stress in Pregnancy. Biomolecules.

[203] Zhang M., Liu Q., Meng H., Duan H., Liu X., Wu J., Gao F., Wang S., Tan R., Yuan J. (2024). Ischemia-reperfusion injury: Molecular mechanisms and therapeutic targets. Signal Transduct. Target. Ther..

[204] Hung T.H., Skepper J.N., Charnock-Jones D.S., Burton G.J. (2002). Hypoxia-reoxygenation: A potent inducer of apoptotic changes in the human placenta and possible etiological factor in preeclampsia. Circ. Res..

[205] Kowalczyk P., Sulejczak D., Kleczkowska P., Bukowska-Ośko I., Kucia M., Popiel M., Wietrak E., Kramkowski K., Wrzosek K., Kaczyńska K. (2021). Mitochondrial Oxidative Stress-A Causative Factor and Therapeutic Target in Many Diseases. Int. J. Mol. Sci..

[206] Xu X., Pang Y., Fan X. (2025). Mitochondria in oxidative stress, inflammation and aging: From mechanisms to therapeutic advances. Signal Transduct. Target. Ther..

[207] Chen S., Li Q., Shi H., Li F., Duan Y., Guo Q. (2024). New insights into the role of mitochondrial dynamics in oxidative stress-induced diseases. Biomed. Pharmacother..

[208] Cipriano A., Viviano M., Feoli A., Milite C., Sarno G., Castellano S., Sbardella G. (2023). NADPH Oxidases: From Molecular Mechanisms to Current Inhibitors. J. Med. Chem..

[209] Annesi L., Tossetta G., Borghi C., Piani F. (2024). The Role of Xanthine Oxidase in Pregnancy Complications: A Systematic Review. Antioxidants.

[210] Botting-Lawford K.J., Skeffington K.L., Murphy M.P., David A.L., Giussani D.A. (2025). Antioxidants: Powering the fight against fetal hypoxia. Philos. Trans. R. Soc. Lond. B Biol. Sci..

[211] Juan C.A., Pérez de la Lastra J.M., Plou F.J., Pérez-Lebeña E. (2021). The Chemistry of Reactive Oxygen Species (ROS) Revisited: Outlining Their Role in Biological Macromolecules (DNA, Lipids and Proteins) and Induced Pathologies. Int. J. Mol. Sci..

[212] Sultana Z., Qiao Y., Maiti K., Smith R. (2023). Involvement of oxidative stress in placental dysfunction, the pathophysiology of fetal death and pregnancy disorders. Reproduction.

[213] Schoots M.H., Gordijn S.J., Scherjon S.A., van Goor H., Hillebrands J.L. (2018). Oxidative stress in placental pathology. Placenta.

[214] Colson A., Sonveaux P., Debiève F., Sferruzzi-Perri A.N. (2021). Adaptations of the human placenta to hypoxia: Opportunities for interventions in fetal growth restriction. Hum. Reprod. Update.

[215] Gautam M., Chaudhary S., Gupta S. (2026). Placental Adaptations Under Severe Anemia: Neonatal Outcomes at the Crossroads. Cureus.

[216] Baker B.C., Heazell A.E.P., Sibley C., Wright R., Bischof H., Beards F., Guevara T., Girard S., Jones R.L. (2021). Hypoxia and oxidative stress induce sterile placental inflammation in vitro. Sci. Rep..

[217] Kumar A., Sharma E., Marley A., Samaan M.A., Brookes M.J. (2022). Iron deficiency anaemia: Pathophysiology, assessment, practical management. BMJ Open Gastroenterol..

[218] Dewey K.G., Oaks B.M. (2017). U-shaped curve for risk associated with maternal hemoglobin, iron status, or iron supplementation. Am. J. Clin. Nutr..

[219] Cariaco Y., Beck M., Jahan F., Kao J.G., Nik-Akhtar A., Menzies K., Bainbridge S. (2025). Ferroptosis as a mechanism of placenta dysfunction in inflammation-driven preeclampsia. Placenta.

[220] Chen W., Tian H., Wei R., Chen X., Jia Y. (2026). Ferritin in ferroptosis: Implications for neurodegenerative diseases (Review). Int. J. Mol. Med..

[221] Chen Y.S., Tian H.X., Rong D.C., Wang L., Chen S., Zeng J., Xu H., Mei J., Wang L.Y., Liou Y.L. (2025). ROS homeostasis in cell fate, pathophysiology, and therapeutic interventions. Mol. Biomed..

[222] Luo T., Wang C., Zhou N., Zhang Y., Mou X. (2026). Lysosomes in Ferroptosis: Regulatory Mechanisms and Molecular Targets. Molecules.

[223] Lennicke C., Cochemé H.M. (2021). Redox metabolism: ROS as specific molecular regulators of cell signaling and function. Mol. Cell..

[224] Fleming A.M., Dingman J.C., Burrows C.J. (2024). CO2 protects cells from iron-Fenton oxidative DNA damage in Escherichia coli and humans. Proc. Natl. Acad. Sci. USA.

[225] Dingman J.C., Fleming A.M., Burrows C.J. (2025). RNA Oxidative Damage by the Iron-Fenton Reaction is Influenced by Bicarbonate Concentration and Ligand Strength. Chem. Res. Toxicol..

[226] Cyr A.R., Domann F.E. (2011). The redox basis of epigenetic modifications: From mechanisms to functional consequences. Antioxid. Redox Signal.

[227] Menezo Y.J., Silvestris E., Dale B., Elder K. (2016). Oxidative stress and alterations in DNA methylation: Two sides of the same coin in reproduction. Reprod. Biomed. Online.

[228] Niu Y., DesMarais T.L., Tong Z., Yao Y., Costa M. (2015). Oxidative stress alters global histone modification and DNA methylation. Free Radic. Biol. Med..

[229] Camarena V., Huff T.C., Wang G. (2021). Epigenomic regulation by labile iron. Free Radic. Biol. Med..

[230] Cox J.L., Talmon G.A., Koepsell S.A. (2016). Human Hemochromatosis Protein (HFE) Immunoperoxidase Stain Highlights Choriocarcinoma within Mixed Germ Cell Tumors. Dis. Markers.

[231] Dixon S.J., Lemberg K.M., Lamprecht M.R., Skouta R., Zaitsev E.M., Gleason C.E., Patel D.N., Bauer A.J., Cantley A.M., Yang W.S. (2012). Ferroptosis: An iron-dependent form of nonapoptotic cell death. Cell.

[232] Liu X., Tuerxun H., Zhao Y., Li Y., Wen S., Li X., Zhao Y. (2025). Crosstalk between ferroptosis and autophagy: Broaden horizons of cancer therapy. J. Transl. Med..

[233] Shen S., Shao Y., Li C. (2023). Different types of cell death and their shift in shaping disease. Cell Death Discov..

[234] Li M., Jin S., Zhu X., Xu J., Cao Y., Piao H. (2024). The role of ferroptosis in central nervous system damage diseases. PeerJ.

[235] Kong Y., Li J., Lin R., Lu S., Rong L., Xue Y., Fang Y. (2024). Understanding the unique mechanism of ferroptosis: A promising therapeutic target. Front. Cell Dev. Biol..

[236] Jin X., Tang J., Qiu X., Nie X., Ou S., Wu G., Zhang R., Zhu J. (2024). Ferroptosis: Emerging mechanisms, biological function, and therapeutic potential in cancer and inflammation. Cell Death Discov..

[237] Chen X., Comish P.B., Tang D., Kang R. (2021). Characteristics and Biomarkers of Ferroptosis. Front. Cell Dev. Biol..

[238] Dixon S.J., Olzmann J.A. (2024). The cell biology of ferroptosis. Nat. Rev. Mol. Cell Biol..

[239] Chen X., Kang R., Kroemer G., Tang D. (2021). Organelle-specific regulation of ferroptosis. Cell Death Differ..

[240] Wu P., Zhang X., Duan D., Zhao L. (2023). Organelle-Specific Mechanisms in Crosstalk between Apoptosis and Ferroptosis. Oxid. Med. Cell Longev..

[241] Costigan A., Hollville E., Martin S.J. (2023). Discriminating Between Apoptosis, Necrosis, Necroptosis, and Ferroptosis by Microscopy and Flow Cytometry. Curr. Protoc..

[242] Mortensen M.S., Ruiz J., Watts J.L. (2023). Polyunsaturated Fatty Acids Drive Lipid Peroxidation during Ferroptosis. Cells.

[243] Forcina G.C., Dixon S.J. (2019). GPX4 at the Crossroads of Lipid Homeostasis and Ferroptosis. Proteomics.

[244] Li W., Liang L., Liu S., Yi H., Zhou Y. (2023). FSP1: A key regulator of ferroptosis. Trends Mol. Med..

[245] Li F.J., Long H.Z., Zhou Z.W., Luo H.Y., Xu S.G., Gao L.C. (2022). System Xc-/GSH/GPX4 axis: An important antioxidant system for the ferroptosis in drug-resistant solid tumor therapy. Front. Pharmacol..

[246] Zhao Y., Qin G., Fan W., Zhang Y., Peng H. (2025). TF and TFRC regulate ferroptosis in swine testicular cells through the JNK signaling pathway. Int. J. Biol. Macromol..

[247] Rochette L., Dogon G., Rigal E., Zeller M., Cottin Y., Vergely C. (2022). Lipid Peroxidation and Iron Metabolism: Two Corner Stones in the Homeostasis Control of Ferroptosis. Int. J. Mol. Sci..

[248] Galluzzi L., Vitale I., Aaronson S.A., Abrams J.M., Adam D., Agostinis P., Alnemri E.S., Altucci L., Amelio I., Andrews D.W. (2018). Molecular mechanisms of cell death: Recommendations of the Nomenclature Committee on Cell Death 2018. Cell Death Differ..

[249] Wan X., Wang X., Liu Y., Hong L., Zhao Z., Guo P. (2025). Ferroptosis in human reproductive tract infections and associated disorders: Mechanisms and emerging therapeutic opportunities. Front. Immunol..

[250] Chabane T., Bouscary D., Grignano E. (2026). NCOA4 and ferritinophagy in hematological malignancies: A double-edged regulator of iron metabolism and cell fate. Front. Oncol..

[251] Santana-Codina N., Gikandi A., Mancias J.D. (2021). The Role of NCOA4-Mediated Ferritinophagy in Ferroptosis. Adv. Exp. Med. Biol..

[252] Wang J., Wu N., Peng M., Oyang L., Jiang X., Peng Q., Zhou Y., He Z., Liao Q. (2023). Ferritinophagy: Research advance and clinical significance in cancers. Cell Death Discov..

[253] Zhao H., Wang Z., Wang H. (2025). The role of NCOA4-mediated ferritinophagy in the ferroptosis of hepatocytes: A mechanistic viewpoint. Pathol. Res. Pract..

[254] Santana-Codina N., Mancias J.D. (2018). The Role of NCOA4-Mediated Ferritinophagy in Health and Disease. Pharmaceuticals.

[255] Jin X., Jiang C., Zou Z., Huang H., Li X., Xu S., Tan R. (2023). Ferritinophagy in the etiopathogenic mechanism of related diseases. J. Nutr. Biochem..

[256] Ning J., Wen L., Qiao L. (2026). Ferritinophagy: Molecular mechanisms and its crosstalk with ferroptosis in chronic respiratory diseases. Cell Biol. Toxicol..

[257] Cariaco Y., Nik-Akhtar A., Brien M.E., Menzies K., Girard S., Bainbridge S. (2026). Placental ferroptosis and impaired fetal growth in symptomatic and asymptomatic SARS-CoV-2 infections. Clin. Sci..

[258] Monisha S., Milan K.L., Anuradha M., Ramkumar K.M. (2026). Ferroptosis-Mediated Placental Dysfunction in the Pathophysiology of Gestational Diabetes Mellitus. Am. J. Reprod. Immunol..

[259] Ng S.W., Lee C., Ng A., Ng S.K., Arcuri F., House M.D., Norwitz E.R. (2023). Ferroportin expression and regulation in human placenta/fetal membranes: Implications for ferroptosis and adverse pregnancy outcomes. Reprod. Biol..

[260] Xu J., Zhou F., Wang X., Mo C. (2023). Role of ferroptosis in pregnancy related diseases and its therapeutic potential. Front. Cell Dev. Biol..

[261] Chen Z., Gan J., Zhang M., Du Y., Zhao H. (2022). Ferroptosis and Its Emerging Role in Pre-Eclampsia. Antioxidants.

[262] Liu J.X., Chen D., Li M.X., Hua Y. (2019). Increased serum iron levels in pregnant women with preeclampsia: A meta-analysis of observational studies. J. Obstet. Gynaecol..

[263] Mo H., Wang Z., Qu C., Liu X. (2025). The associations of maternal serum ferritin levels with hypertensive disorders of pregnancy: A longitudinal cohort study. Front. Nutr..

[264] Beharier O., Kajiwara K., Sadovsky Y. (2021). Ferroptosis, trophoblast lipotoxic damage, and adverse pregnancy outcome. Placenta.

[265] Torres-Torres J., Espino-Y-Sosa S., Martinez-Portilla R., Borboa-Olivares H., Estrada-Gutierrez G., Acevedo-Gallegos S., Ruiz-Ramirez E., Velasco-Espin M., Cerda-Flores P., Ramirez-Gonzalez A. (2024). A Narrative Review on the Pathophysiology of Preeclampsia. Int. J. Mol. Sci..

[266] Park C., Alahari S., Ausman J., Liu R., Nguyen F., Sallais J., Post M., Caniggia I. (2025). Placental Hypoxia-Induced Ferroptosis Drives Vascular Damage in Preeclampsia. Circ. Res..

[267] Wrighting D.M., Andrews N.C. (2006). Interleukin-6 induces hepcidin expression through STAT3. Blood.

[268] Bandyopadhyay A., Ahamed F., Palepu S., Ghosh T., Yadav V. (2022). Association of Serum Hepcidin With Preeclampsia: A Systematic Review and Meta-Analysis. Cureus.

[269] Shaji Geetha N., Bobby Z., Dorairajan G., Jacob S.E. (2022). Increased hepcidin levels in preeclampsia: A protective mechanism against iron overload mediated oxidative stress?. J. Matern. Fetal Neonatal Med..

[270] Aires Rodrigues de Freitas M., Vieira da Costa A., Alves de Medeiros L., da Silva Garrote Filho M., Lemos Debs Diniz A., Penha-Silva N. (2018). Are There Differences in the Anthropometric, Hemodynamic, Hematologic, and Biochemical Profiles between Late- and Early-Onset Preeclampsia?. Obstet. Gynecol. Int..

[271] Ahmed Y.I.B., Yagoub H.S., Hassan M.A., Adam I., Hamdan H.Z. (2023). Maternal serum iron status, hepcidin and interleukin-6 levels in women with preeclampsia. Front. Physiol..

[272] Brunacci F., Rocha V.S., De Carli E., Espósito B.P., Ruano R., Colli C. (2018). Increased serum iron in preeclamptic women is likely due to low hepcidin levels. Nutr. Res..

[273] Kornacki J., Olejniczak O., Sibiak R., Gutaj P., Wender-Ożegowska E. (2023). Pathophysiology of Pre-Eclampsia-Two Theories of the Development of the Disease. Int. J. Mol. Sci..

[274] Yang X., Ding Y., Sun L., Shi M., Zhang P., Huang Z., Wang J., He A., Wang J., Wei J. (2022). Ferritin light chain deficiency-induced ferroptosis is involved in preeclampsia pathophysiology by disturbing uterine spiral artery remodelling. Redox Biol..

[275] Geng H., Zhang P., Zhou L., Chen X. (2026). MiR-150-3p as a diagnostic biomarker and regulator of ferroptosis in preeclampsia via targeting FTH1. Cytotechnology.

[276] Zhang H., He Y., Wang J.X., Chen M.H., Xu J.J., Jiang M.H., Feng Y.L., Gu Y.F. (2020). miR-30-5p-mediated ferroptosis of trophoblasts is implicated in the pathogenesis of preeclampsia. Redox Biol..

[277] Jaszczuk I., Koczkodaj D., Kondracka A., Kwaśniewska A., Winkler I., Filip A. (2022). The role of miRNA-210 in pre-eclampsia development. Ann. Med..

[278] Monisha S., Milan K.L., Ramkumar K.M. (2026). Death by Iron: Ferroptosis in the Aetiology and Outcome of Gestational Diabetes Mellitus. Diabetes Metab. Res. Rev..

[279] Katsi V., Alifragki A., Fragkiadakis K., Kopidakis N., Kallergis E., Zacharis E., Kampanieris E., Simantirakis E., Tsioufis K., Marketou M. (2025). The Emerging Roles of Ferroptosis and NETosis in Pregnancy Complications: Insights into Preeclampsia and Gestational Diabetes Mellitus. Curr. Issues Mol. Biol..

[280] Tossetta G., Campagna R., Vignini A., Maruotti G.M., Motta M., Murolo C., Sarno L., Grelloni C., Cecati M., Giannubilo S.R. (2026). The Placenta in Gestational Diabetes: An Integrated Review on Metabolic Pathways, Genetic, Epigenetic and Ultrasound Biomarkers for Clinical Perspectives. Int. J. Mol. Sci..

[281] Zgutka K., Tkacz M., Tomasiak P., Piotrowska K., Ustianowski P., Pawlik A., Tarnowski M. (2024). Gestational Diabetes Mellitus-Induced Inflammation in the Placenta via IL-1β and Toll-like Receptor Pathways. Int. J. Mol. Sci..

[282] Pan X., Jin X., Wang J., Hu Q., Dai B. (2021). Placenta inflammation is closely associated with gestational diabetes mellitus. Am. J. Transl. Res..

[283] Li J., Su X., Wei W., Xu Y., Ni Y., Baimu D., Yan H., Yang H., Ye H. (2026). Association of iron nutritional homeostasis and hepcidin gene polymorphisms with gestational diabetes mellitus. BMC Pregnancy Childbirth.

[284] Kato J., Kobune M., Ohkubo S., Fujikawa K., Tanaka M., Takimoto R., Takada K., Takahari D., Kawano Y., Kohgo Y. (2007). Iron/IRP-1-dependent regulation of mRNA expression for transferrin receptor, DMT1 and ferritin during human erythroid differentiation. Exp. Hematol..

[285] Georgieff M.K., Berry S.A., Wobken J.D., Leibold E.A. (1999). Increased placental iron regulatory protein-1 expression in diabetic pregnancies complicated by fetal iron deficiency. Placenta.

[286] Yang A., Zhao J., Lu M., Gu Y., Zhu Y., Chen D., Fu J. (2016). Expression of Hepcidin and Ferroportin in the Placenta, and Ferritin and Transferrin Receptor 1 Levels in Maternal and Umbilical Cord Blood in Pregnant Women with and without Gestational Diabetes. Int. J. Environ. Res. Public Health.

[287] Zaugg J., Melhem H., Huang X., Wegner M., Baumann M., Surbek D., Körner M., Albrecht C. (2020). Gestational diabetes mellitus affects placental iron homeostasis: Mechanism and clinical implications. FASEB J..

[288] Huang Z., Zhou S., Cui Z., Li J., Liu L., Feng X., Ye H., Ma K., Huang S., Xiang Q. (2026). DHA ameliorates GDM placental angiogenesis by regulating HAMP-mediated iron imbalance. J. Adv. Res..

[289] Liang X., Zhang J., Wang Y., Wu Y., Liu H., Feng W., Si Z., Sun R., Hao Z., Guo H. (2023). Comparative study of microvascular structural changes in the gestational diabetic placenta. Diab Vasc. Dis. Res..

[290] Khodaei M.M., Noori Z., Zare F., Meshkin A. (2025). Ferroptosis and recurrent miscarriage: A critical review of pathophysiology and emerging therapeutic targets. Front. Cell Dev. Biol..

[291] Yoshida K., Kusama K., Sato S., Kawaguchi Y., Horiuchi A., Yoshie M., Tamura K. (2025). Role of ferroptosis-related GPX4 signaling in the fusion of human trophoblast cells. Sci. Rep..

[292] Sun Y., Sha M., Qin Y., Xiao J., Li W., Li S., Chen S. (2024). Bisphenol A induces placental ferroptosis and fetal growth restriction via the YAP/TAZ-ferritinophagy axis. Free Radic. Biol. Med..

[293] Qiu L., Liu H., Chen S., Wu Y., Yan J. (2025). Ferroptosis contributed to endoplasmic reticulum stress in preterm birth by targeting LHX1 and IRE-1. Cell Signal.

[294] Liu C., Zhang Q., Hui P., Wang Y., Li G., Cao G., Xue Z., Zhang J., Zhang H., Huang X. (2023). Safety monitoring of oral iron supplements in pregnant women with anemia: A multi-center observational clinical study. Ther. Adv. Drug Saf..

[295] Zaugg J., Lopez-Tello J., Musial B., Vaughan O.R., Fowden A.L., Albrecht C., Sferruzzi-Perri A.N. (2024). Obesogenic diet in pregnancy disrupts placental iron handling and ferroptosis and stress signalling in association with fetal growth alterations. Cell Mol. Life Sci..

[296] Doshani A., Konje J.C. (2023). Placental dysfunction in obese women and antenatal surveillance. Best. Pract. Res. Clin. Obstet. Gynaecol..

[297] Yan Y., Guo F., Liu K., Ding R., Wang Y. (2023). The effect of endocrine-disrupting chemicals on placental development. Front. Endocrinol..

[298] Gong F.H., Liu L., Wang X., Xiang Q., Yi X., Jiang D.S. (2025). Ferroptosis induced by environmental pollutants and its health implications. Cell Death Discov..

[299] Xie Y., Li C., Dong X., Wang B., Qin J., Lv H. (2025). Natural Products as Modulators of Iron Metabolism and Ferroptosis in Diabetes and Its Complications. Nutrients.

[300] Di Giacomo C., Malfa G.A., Tomasello B., Bianchi S., Acquaviva R. (2023). Natural Compounds and Glutathione: Beyond Mere Antioxidants. Antioxidants.

[301] Du X., Elsabagh M., He F., Wu H., Zhang B., Fan K., Wang M., Zhang H. (2025). Gut Microbiota and Its Metabolites Modulate Pregnancy Outcomes by Regulating Placental Autophagy and Ferroptosis. Antioxidants.

[302] Zhang M., Chen X., Zhang Y. (2024). Mechanisms of Vitamins Inhibiting Ferroptosis. Antioxidants.

[303] Wu J., Shi Y., Zhou M., Chen M., Ji S., Liu X., Zhou M., Xia R., Zheng X., Wang W. (2024). Nutrient vitamins enabled metabolic regulation of ferroptosis via reactive oxygen species biology. Front. Pharmacol..

[304] Chen Y., Li X., Wang S., Miao R., Zhong J. (2023). Targeting Iron Metabolism and Ferroptosis as Novel Therapeutic Approaches in Cardiovascular Diseases. Nutrients.

[305] Prabhune N.M., Ameen B., Prabhu S. (2025). Therapeutic potential of synthetic and natural iron chelators against ferroptosis. Naunyn Schmiedebergs Arch. Pharmacol..

[306] Zilka O., Shah R., Li B., Friedmann Angeli J.P., Griesser M., Conrad M., Pratt D.A. (2017). On the Mechanism of Cytoprotection by Ferrostatin-1 and Liproxstatin-1 and the Role of Lipid Peroxidation in Ferroptotic Cell Death. ACS Cent. Sci..

[307] Miotto G., Rossetto M., Di Paolo M.L., Orian L., Venerando R., Roveri A., Vučković A.M., Bosello Travain V., Zaccarin M., Zennaro L. (2020). Insight into the mechanism of ferroptosis inhibition by ferrostatin-1. Redox Biol..

[308] Tong J., Lan X.T., Zhang Z., Liu Y., Sun D.Y., Wang X.J., Ou-Yang S.X., Zhuang C.L., Shen F.M., Wang P. (2023). Ferroptosis inhibitor liproxstatin-1 alleviates metabolic dysfunction-associated fatty liver disease in mice: Potential involvement of PANoptosis. Acta Pharmacol. Sin..

[309] Zhuang X., Shi S., Liu S., Jiao Y., Huang B., Yang Y., Yang L., Yang X., Wang H., Liang C. (2025). Dipyridamole Acts as Clinical Ferroptosis Inhibitor to Prevent from Tissue Injury. Adv. Sci..

[310] Lianski S., Mizrachi T., Barak O., Tsaitlin-Mor L., Elhaik-Goldman S., Yagel S., Goldman-Wohl D., Cohen S.M., Hefetz Medina R., Rachmilewitz J. (2025). Antiferroptotic Drugs Reduce sFlt-1 Release and Placenta Damage in Preeclampsia. Hypertension.

[311] Jiang Y., Glandorff C., Sun M. (2024). GSH and Ferroptosis: Side-by-Side Partners in the Fight against Tumors. Antioxidants.

[312] Beharier O., Tyurin V.A., Goff J.P., Guerrero-Santoro J., Kajiwara K., Chu T., Tyurina Y.Y., St Croix C.M., Wallace C.T., Parry S. (2020). PLA2G6 guards placental trophoblasts against ferroptotic injury. Proc. Natl. Acad. Sci. USA.

[313] Ngo V., Duennwald M.L. (2022). Nrf2 and Oxidative Stress: A General Overview of Mechanisms and Implications in Human Disease. Antioxidants.

[314] Tantengco O.A.G., de Castro Silva M., Shahin H., Bento G.F.C., Cursino G.C., Cayenne S., da Silva M.G., Menon R. (2021). The role of nuclear factor erythroid 2-related factor 2 (NRF2) in normal and pathological pregnancy: A systematic review. Am. J. Reprod. Immunol..

[315] Li Y.Q., Yan H., Bai B. (2008). Change in iron transporter expression in human term placenta with different maternal iron status. Eur. J. Obstet. Gynecol. Reprod. Biol..

